# Methodological considerations in studying digestive system physiology in octopus: limitations, lacunae and lessons learnt

**DOI:** 10.3389/fphys.2022.928013

**Published:** 2022-09-09

**Authors:** Paul L. R. Andrews, Giovanna Ponte, Carlos Rosas

**Affiliations:** ^1^ Department of Biology and Evolution of Marine Organisms, Stazione Zoologica Anton Dohrn, Naples, Italy; ^2^ Unidad Multidisciplinaria de Docencia e Investigación, Facultad de Ciencias, Universidad Nacional Autónoma de México, Sisal, Yucatán, Mexico

**Keywords:** octopus, digestive tract, digestive gland, motility, digestion, secretion, welfare, Directive 2010/63/EU

## Abstract

Current understanding of cephalopod digestive tract physiology is based on relatively “old” literature and a “mosaic of data” from multiple species. To provide a background to the discussion of methodologies for investigating physiology we first review the anatomy of the cephalopod digestive tract with a focus on *Octopus vulgaris*, highlighting structure-function relationships and species differences with potential functional consequences (e.g., absence of a crop in cuttlefish and squid; presence of a caecal sac in squid). We caution about extrapolation of data on the digestive system physiology from one cephalopod species to another because of the anatomical differences. The contribution of anatomical and histological techniques (e.g., digestive enzyme histochemistry and neurotransmitter immunohistochemistry) to understanding physiological processes is discussed. For each major digestive tract function we briefly review current knowledge, and then discuss techniques and their limitations for the following parameters: *1*) Measuring motility *in vitro* (e.g., spatiotemporal mapping, tension and pressure), *in vivo* (labelled food, high resolution ultrasound) and aspects of pharmacology; *2*) Measuring food ingestion and the time course of digestion with an emphasis on understanding enzyme function in each gut region with respect to time; *3*) Assessing transepithelial transport of nutrients; *4*) Measuring the energetic cost of food processing, impact of environmental temperature and metabolic rate (flow-through/intermittent respirometry); *4*) Investigating neural (brain, gastric ganglion, enteric) and endocrine control processes with an emphasis on application of molecular techniques to identify receptors and their ligands. A number of major knowledge lacunae are identified where available techniques need to be applied to cephalopods, these include: *1*) What is the physiological function of the caecal leaflets and intestinal typhlosoles in octopus? *2*) What role does the transepithelial transport in the caecum and intestine play in ion, water and nutrient transport? *3*) What information is signalled from the digestive tract to the brain regarding the food ingested and the progress of digestion? It is hoped that by combining discussion of the physiology of the cephalopod digestive system with an overview of techniques and identification of key knowledge gaps that this will encourage a more systematic approach to research in this area.

## Introduction

The digestive system (DS) in cephalopods includes an epithelium-lined muscular tract (DT), and glands (salivary glands and digestive gland [DG]± appendages). The DS physically and chemically degrades food, absorbs nutrients and processes them for metabolism. The DG has a major role in detoxification of ingested potential toxins (e.g., domoic acid, metals) but the “barrier function” of the epithelium also contributes to defence of the organism. The DS is also responsible for excretion of waste products (faeces, including mucus and sloughed epithelial cells) and voiding of undigested and indigestible matter (i.e. faeces, vomit).

Studying the physiology of the cephalopod DS can be justified from a pure science perspective, particularly contributing to comparative and evolutionary studies. For example, comparison with fish as the main predators of cephalopods in the same ecological niches has been considered mainly from a brain evolution perspective (e.g., Ponte et al., 2021), but this should be broadened to include the DS which processes food to “fuel” brain tissue. In “Cephalopods and fish: The limits of convergence,” Packard (1972) commented on similarities in relation to the caecum but other comparisons between fish and cephalopod digestive tracts are unexplored. There are also applied research justifications to consider: *1*) Optimising diets at all life stages in aquaculture; *2*) Understanding the consequences of dietary change in the wild; *3*) Understanding adaptations to environmental change; *4*) Assessing the impact of ingestion of plastics and other contaminants on the digestive tract, health and welfare; *5*) Animal welfare in the laboratory, public display and aquaculture (*see* Sykes et al., 2017, for detailed discussion).

This review considers techniques used to study the physiological processes underlying key functions of the digestive system in cephalopods with a particular focus on octopuses as these have been the subject of most recent studies.

To provide a background we briefly describe the range of food eaten by cephalopods and then the anatomy of the cephalopod DT highlighting major species differences.

## The range of substances ingested: challenges for the digestive system

Villanueva et al. (2017) commented that food sources ranged from “detritus to birds.” All cephalopods are carnivorous with cephalopods, fish, gastropods and crustacea amongst the most common foods depending upon the species and habitat. Chemically, the diet is dominated by proteins and lipids. However, field studies have widened the range of food types to include for example, gelatinous fauna by the giant deep-sea octopus (*Haliphron atlanticus*, Hoving and Haddock, 2017) and various types of detritus (e.g., faecal pellets, gelatinous zooplankton) by vampire squid (*Vampyroteuthis infernalis*, Hoving and Robison, 2012).

The digestive system also deals with non-prey items. Examples include, microplastics in the water, adherent to, or ingested by the prey. Evidence for microplastic ingestion has been published for wild and cultured cuttlefish (*Sepia officinalis*, Oliveira et al., 2020), common octopus (*Octopus vulgaris*, Pedà et al., 2022), jumbo squid (*Dosidicus gigas*: Braid et al., 2012; Rosas-Luis, 2016), vampire and mid-water squid (*V. infernalis* and *Abralia veranyi*, Ferreira et al., 2022). Although no adverse effects are reported, the potential for harm, particularly from obstruction of the ducts linking the caecum and digestive gland is considerable (for discussion *see* Oliveira et al., 2020). Plant matter (e.g., seaweed, bull kelp, eelgrass) has been found in the cephalopod digestive tract (Boucaud-Camou and Boucher-Rodoni, 1983; Braid et al., 2012). In theory, non-prey items could be digested by enzymes, ejected unchanged by defaecation or vomiting/regurgitation or remain in the tract where, if accumulated, they could cause an obstruction.

Food ingestion will be accompanied by seawater, potentially containing dissolved or suspended chemical contaminants. The first report of metals in the cephalopod DG was in 1975 (Martin and Flegal, 1975; Bustamante et al., 1998) with high levels of cadmium, copper, iron, silver and zinc in *Loligo opalescens*, *Ommastrephes bartrami*, and *Sthenoteuthis oualaniensis*. The metal concentrations were considered sufficient to be toxic. Bustamante et al. (2006) reviewed the DG and muscle mercury content in 20 cephalopod species concluding that the DG did not store mercury, but demethylated it allowing accumulation in organic form in muscle. Rodrigo and Costa (2017) identified the DG as the main metal accumulation organ and concluded that metallothionines were not the only detoxification mechanism with spherulae (high MWt. protein and metal association) chelating metals (*see* also, Bustamante et al., 2006). B-esterases have recently been implicated in metabolism of plastic additives in the DG of *O. vulgaris* and *S. officinalis* (Omedes et al., 2022).

The biochemical pathways involved in processing metals and other potentially toxic chemicals (e.g., domoic acid) in the DG, their subsequent distribution to other tissues (e.g., brain, muscle) and the functional consequences (e.g., impaired metabolism, modified neuronal function) require further study, especially considering the important role of cephalopods in the marine ecosystems.

Sea water drinking occurs in marine teleosts and elasmobranchs and is an important component of fluid and ionic homeostasis (for review *see*: Grosell, 2010; Takei, 2021). Haemolymph osmolarity measured in *O. vulgaris, O. insularis* and *O. ocellatus* ranged from ∼940 to ∼1170 mOsm/kg (Amado et al., 2015; Sakamoto et al., 2015) so is slightly hypo-osmotic or iso-osmotic with sea water. The values are comparable to marine osmoconforming fish but considerably higher than the osmoregulating teleosts (Takei, 2021). Wells and Wells (1989; 1993) investigated water uptake in *O. vulgaris* and concluded that the digestive gland appendage (“pancreas”) is one site of fluid and ion transport. Of relevance to this is the finding in squid (*L. vulgaris*) and cuttlefish (*S. officinalis*) hatchlings (Hu et al., 2010) that the appendage shows Na^+^/K^+^ ATPase immunoreactivity; this is significant as this ion exchange pump is responsible for generating the local osmotic gradient in the lateral intercellular spaces responses for transepithelial water transport but there are no physiological studies of fluid/ionic transport in cephalopod gut. Wells and Wells (1989, p. 219) describe rectal ingestion of sea water in *O. vulgaris* and Mangold and Young (1998) include “intake of seawater” in their list of intestinal functions in cephalopods. Furthermore, Bidder (1950) reports that water is drawn into the intestine of “larval” *L. vulgaris* and that this may occur in adults monitored by the processes of specialised rectal cells and involving the anal leaflets. These latter observations require investigation using modern physiological techniques.

## A brief introduction to the anatomy of the cephalopod digestive system: implications for physiology

The structure and function of organs is related. As a background, we briefly describe the anatomy of the digestive tract in *O. vulgaris*, highlighting poorly understood aspects in relation to function and then discuss the main anatomical differences from other cephalopods to emphasise the necessity to study a variety of species.

Table 1 summarises publications describing the gross anatomy of the digestive system in a range of cephalopod species and also provides references to specific topics of relevance to understanding physiology.

**TABLE 1:** Selected key publications that include detailed descriptions of the anatomy and histology of the digestive system and its innervation in a range of cephalopod species.

Species	Comment	References
**Gross anatomy of the digestive system in adults**		
*Loligo vulgaris, Loligo forbesii, Alloteuthis media, Alloteuthis subulata*	Detailed description of *Loligo vulagris* and comments on other species. Summary of pre-1930s literature	Bidder (1950)
*Loligo vulgaris*	Monograph on the common squid including description of the digestive system and feeding	Williams (1909)
*Eledone cirrhosa*	Monograph with very detailed diagrams of the digestive system	Isgrove (1909)
*Octopus vulgaris*, *Abralia trigonura*, *Loligo vulgaris*, *Vampyroteuthis infernalis*	Diagrams of gross anatomy and a tabulated comparison of digestive organs between Nautloidoea and several species of Coleoidea	Mangold and Young (1998)
*Todarodes pacificus*, *Loligo bleekeri*, *Loligo edulis*, *Watasenia scintillans*, *Sepia lycidas, Euprymna morsei*	Diagrams of gross anatomy of six Decapodiform cephalopods and data on some organ weights	Omura and Endo (2016)
*Octopus americanus*	Diagram and brief description of digestive tract	Avendaño et al. (2020)
*Nautilus pompilius*	Original description of external and internal anatomy of Nautilus	Owen (1832)
		Westermann and Schipp (1998b), also comments on *Nautilus macromphalus*
*Sepia officinalis*	Monograph with very detailed diagrams of the digestive system	Tompsett (1939)
*Enteroctopus megalocyathus*	Basic description and photograph of digestive tract	Garri and Lauria de Cidre (2013)
*Cirrothauma*	Diagram and description of digestive tract in a finned octopod	Aldred et al. (1983)
*Sepia officinalis, Octopus vulgaris, Loligo vulgaris*	General description of anatomy and importantly a photograph of the tract in each species	Guerra (2019)
**Gross anatomy of the digestive system in paralarvae**		
*Octopus vulgaris*	3D reconstruction during the first month of life and also data on embryos	Fernández-Gago et al. (2017)
*Octopus vulgaris*	Detailed colour images of the digestive tract in live feeding paralarvae taking advantage of their transparency at this life stage	Nande et al. (2017)
**Histology of the digestive tract**		
*Enteroctopus megalocyathus*	Sections of several regions but particularly the caecum	Garri and Lauria de Cidre (2013)
*Octopus vulgaris*	Haematoxylin & Eosin stained section of several regions	Emam et al. (2016)
*Nautilus pompilius* on *Nautilus macromphalus*	Sections from all main regions and also scanning electron microscopy of caecum	Westermann and Schipp (1998b)
*Octopus vulgaris*	Detailed survey of all regions of the digestive tract	Fernández-Gago et al. (2019)
*Octopus vulgaris*	Detailed survey of all regions of the digestive tract	Anadón (2019)
*Loligo vulgaris, Loligo forbesii, Alloteuthis media, Alloteuthis subulata*	Histology of epithelium in oesophagus stomach and caecum and diagrammatic reconstructions of caecal wall	Bidder (1950)
*Eledone cirrosa Illex illecebrosus*	Study of histological changes occurring during the course of digestion	Boucher-Rodoni (1976)
**Histopathology of the digestive tract**		
*Octopus vulgaris*	Histopathology changes in the caecum and intestine caused by *Aggregata octopiana*	Gestal et al. (2002)
Multiple species	Comprehensive review of pathogens and diseases including those affecting the digestive tract	Gestal et al. (2019)
**Histology of digestive gland (DG)**		
*Octopus vulgaris*		Rodrigo and Costa (2017)
		Fernández-Gago et al. (2019)
*Enteroctopus mergalocythus*		Garri and Lauria de Cidre (2013)
*Sepia officinalis*		Costa et al. (2014)
*Nautilus pompilius L. and Nautilus macromphalus*		Ruth et al. (1999)
*Euprymna tasmanica* (dumpling squid)	Investigation of potential lipid storage in DG.	Moltschaniwskyj and Johnston (2006)
**Enzyme and mucus histochemistry**		
*Octopus vulgaris*	Detailed survey of mucus and granule secreting cells in all regions of the tract	Fernández-Gago et al. (2019)
**Innervation of the digestive system**		
*Eledone cirrhosa*	Detailed diagrams of the gastric ganglion and associated nerves	Isgrove (1909)
*Octopus vulgaris*	Detailed description of the extrinsic (visceral and sympathetic) innervation	Young (1967; 1971)
	Molecular study of the neurochemistry of the gastric ganglion	Baldascino et al. (2017)
*Sepia officinalis*	Focus on the nerve plexuses within the wall of the digestive tract	Alexandrowicz (1928)

### The anatomy of the digestive tract in *O. vulgaris*: relationships to physiology

The buccal mass comprising the beak, radula and associated muscles is the point at which food enters the body with the size of the bolus determined by the gape of the beak and the bite force. The food is mixed with secretions from the anterior and posterior salivary glands which contain digestive enzymes but also in the case of the posterior glands toxins used to subdue or kill prey (*see* below for details and Ponte and Modica, 2017).

From the buccal mass, pieces of food are propelled along the cuticle lined oesophagus which passes between the sub- and supra-oesophageal lobes of the brain linked by the connectives. The distensibility of the oesophagus and the encircling brain tissue will limit the bolus size together with the compressibility of the food by the oesophageal muscle which in its proximal part has functional characteristics of striated muscle (Andrews and Tansey, 1983a).

**FIGURE 1:**
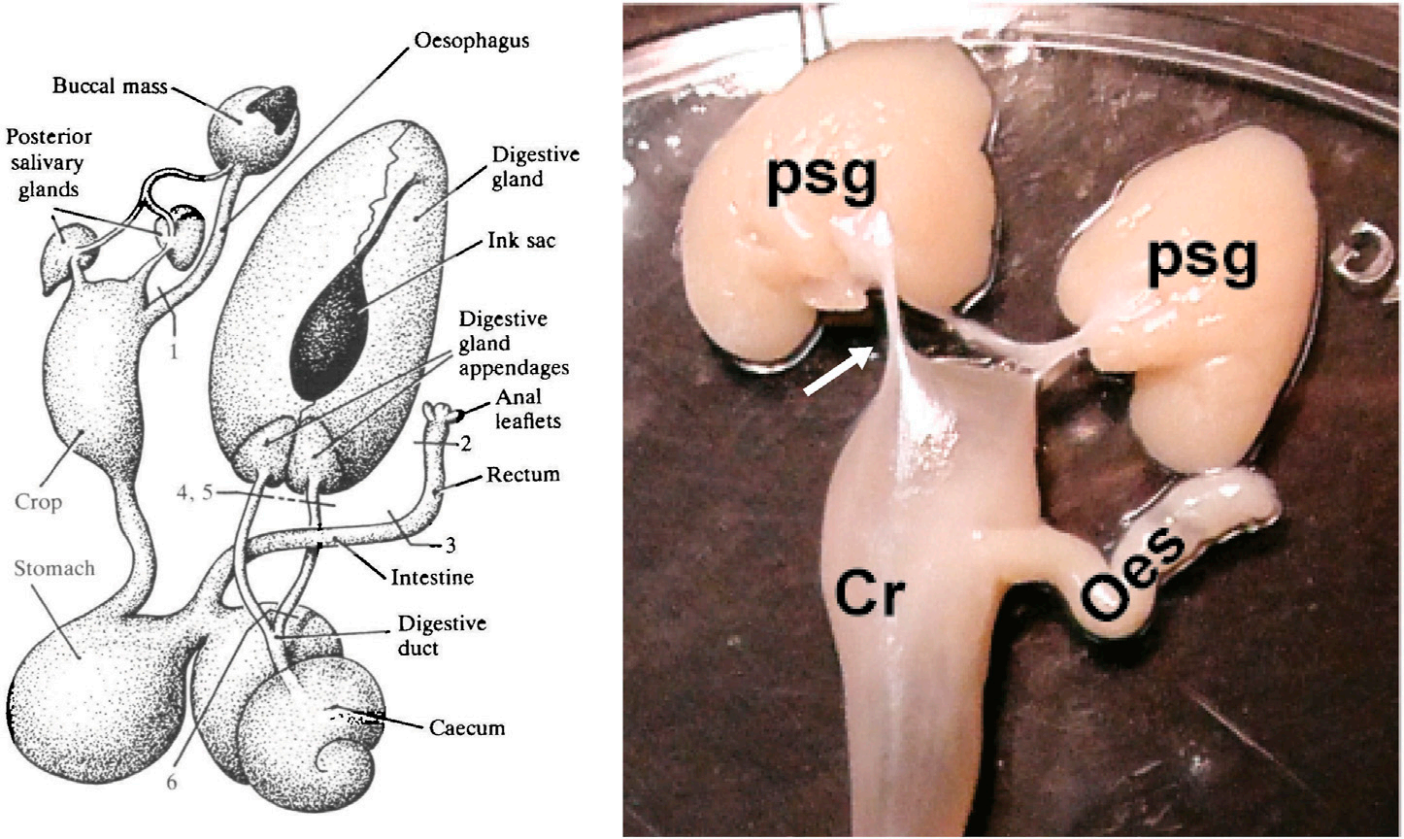
The diagram on the left shows the digestive tract of *Octopus vulgaris*. Note that the posterior salivary glands are connected to the buccal mass by a duct which delivers saliva containing digestive enzymes and toxins for injection into prey and each gland is also connected to the crop by a duct-like structure (*see* text for discussion). The labelling of the original is retained with the numbers indicating sites at which the tract was lesioned/ligated for studies of water uptake. Reproduced from Wells and Wells, 1989, p.217 with permission from The Company of Biologists, Cambridge, United Kingdom. The photograph on the right is taken from Baldascino et al., 2017 (Figure 1) and shows the posterior salivary glands (psg) in *Octopus vulgaris* and the connections (arrowed only on the left) to the anterior crop (Cr). The oesophagus (oes) detached from the buccal mass is also visible.

Following passage along the oesophagus, the bolus enters the crop with very limited evidence for a sphincter between the two structures (for details *see* Sykes et al., 2020). Diagrams of the digestive tract in *O. vulgaris* (*see* Figure 1) sometimes show a bridge of tissue linking each posterior salivary gland to the anterior crop and this can also be seen in published pictures (for *O. vulgaris see* fig. 1 in Baldascino et al., 2017; for *O. americanus see* fig. 3 in Avendaño et al., 2020). Although this structure appears like a duct transporting salivary secretions into the crop, this has not been demonstrated (G. Ponte and P.L.R. Andrews, unpublished observations). Studies of this structure should be undertaken in paralarvae to investigate patency as the structure in adults may be vestigial. Another possibility is that the connexion serves to hold the salivary glands in position as the crop fills.

**FIGURE 2:**
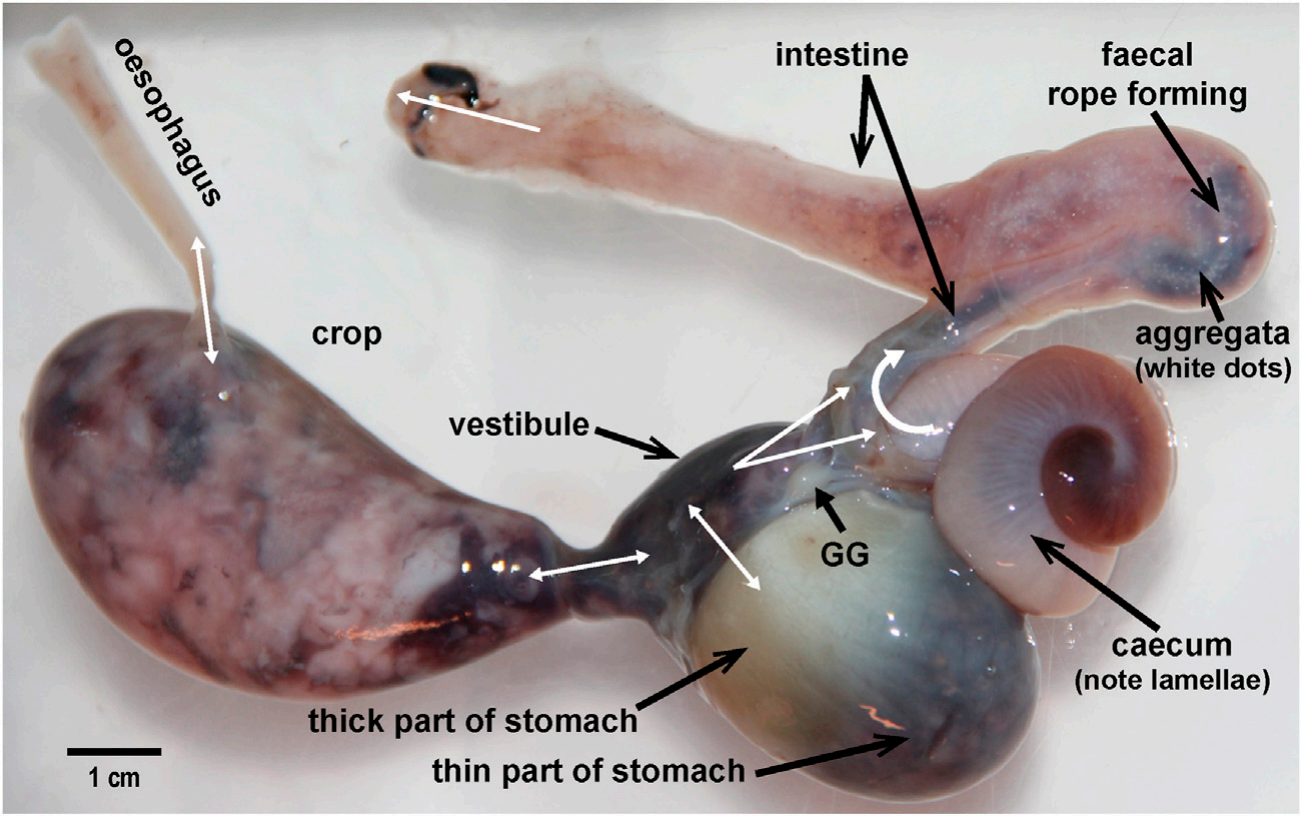
The digestive tract removed from an *Octopus vulgaris* weighing 900 g. The main anatomical regions are labelled including the gastric ganglion (GG). Double headed white arrows indicate the potential for bi-directional exchange of material due the existence of a common cavity. For the anal opening although only unidirectional flow (defaecation) is indicated there is some evidence for the entry of sea water *via* this route (*see* text for discussion). Note the “teardrop” shape of the crop distended with food (primarily an octopus arm), the relatively thin and thick parts of the stomach, the caecal lamellae visible through the thin wall, the presence of aggregata in the intestine, and the location of the GG with nerve radiating to adjacent structures.

When distended with food, the crop can be seen to have a more distensible anterior half (*see* Figure 2) which accommodates ∼75% of the content and a narrower posterior part connecting to the gastric vestibule (Andrews and Tansey, 1983a) with no strong evidence for a sphincter. The gastric vestibule is one of two thin layers of muscle holding the two thick muscle blocks closely apposed and responsible for the movement of one thick block against the other. The stomach has a relatively thick cuticle lining. The anatomical similarity of the stomach in octopus (and to a lesser extent cuttlefish) to that of the koilen-lined “gizzard” present in some species of bird was first noted by Aristotle (1910). The gastric vestibule connects with both the spiral caecum and the intestine with evidence for sphincters between these structures lacking. The major enzymes responsible for digestion (*see* below) originate in the digestive gland, are passed into the caecum (DG ducts) from where they can enter the gastric vestibule to contact the food. The capacity of the stomach in octopus is small in relation to the crop so only relatively small boluses of food can enter for trituration while enzymes continue to act and then the partially digested food progresses to the either caecum or intestine. Alternatively, the crop and stomach may act as a single functional unit with food interchanged between the “hopper” (crop) and “mill” (stomach) until it is physically and chemically degraded and is passed to the caecum or intestine. Currently, there is no definitive evidence to decide between the two, not incompatible, options. The common cavity between the crop, stomach, caecum and intestine means that contents can readily interchange depending upon the intraluminal pressure gradient generated by the muscles in each region.

In *O. vulgaris*, the oesophagus, crop and particularly the stomach are all lined with a cuticle. The cuticle protects the underlying epithelium against abrasion by ingested prey (e.g., crab carapace and limbs, fish bones) and in the stomach provides a surface for trituration. The presence of a continuous, variable thickness, layer of cuticle makes it highly unlikely that the oesophagus, crop or stomach has an absorptive function. However, it should be noted that limited evidence has been provided for uptake of radio-labelled glycine/leucine by the crop in *Nautilus pompilius* (Westermann and Schipp, 1998a) and *O. vulgaris* (Wells, 1978) but uptake into the epithelium does not provide evidence for transepithelial transport.

The caecum is a thin-walled muscular tube, coiled into about two and a half turns forming a cone and lined with lamellae which will increase the surface area. It is stated that the caecum filters food from the stomach “discarding gross particles that pass directly to the intestine” (Anadón, 2019, p. 4). Whilst this is a likely function, together with the addition of mucus to begin formation of faecal ropes, there are no direct studies in octopus demonstrating this but studies of the caecum in squid (Bidder, 1950) are supportive. Additionally, cilia have been to proposed to be responsible for the movement of “fine particles in suspension” from the caecum into the digestive gland (Fernández-Gago et al., 2019, p. 331) but no studies have investigated the relative roles of the cilia, caecal muscle constricting the cone or DG duct peristalsis in this process. The large surface area of the caecum is also consistent with an absorptive function, but Fernández-Gago et al. (2019, p. 331) concluded that in *O. vulgaris* that there was “no clear evidence for absorption in the caecum” although Boucaud-Camou et al. (1976) considered it was involved in absorption as is also the case in *N. pompilius* (Westermann and Schipp, 1998b). In the squid the caecum/caecal sac is proposed to have a major role in absorption (Boucher-Rodoni and Mangold, 1977; Boucaud-Camou and Boucher-Rodoni, 1983). Published studies used uptake of radiolabelled molecules by the epithelium or the presence of lipid containing vesicles in the epithelium but have not demonstrated transepithelial transport into the haemolymph from the caecum and this is therefore a major knowledge gap.

The control of the directionality of movement of contents between the common cavities of the crop, stomach and vestibule, caecum (and hence the DG duct) and proximal intestine has not been investigated. Evidence for the presence of sphincters is inconclusive or absent in octopus although there may be a flap-valve between the caecum and the digestive gland duct at the tip of the columella (Budelmann et al., 1997; *see* figure 64A, p. 197) and a thickening of circular muscle at the exit of the ink duct and anus is consistent with it acting as a sphincter (Young, 1967).

The tubular intestine shows little external variation in octopus throughout its course from the stomach vestibule/caecal junction to the anal opening, but turns anteriorly in the middle of its course. Internally, the mucosa has longitudinal folds, two of which on opposite sides are larger forming a “typhlosole” in the proximal intestine. It is assumed that the function of the typhlosole is to increase surface area and whilst this is a logical assumption the question of the reason why a surface area increase is required has not been answered; usually this is to facilitate absorption but it could also be related to secretory capacity (e.g., mucus). It is reported that the epithelium of the typhlosoles is ciliated but that of adjacent intestinal epithelial tissue has a luminal border with microvilli (Anadón, 2019; Fernández-Gago et al., 2019) suggesting a differentiation of function. Ciliary motion would be capable of moving mucus with trapped particles so investigation of the direction of ciliary beating in fresh specimens would be of particular interest. The function of the typhlosoles is not established, but constriction of the circular muscle would lead to the division of the lumen into two channels by apposition of the typhlosoles. A further issue regarding intestinal function in octopus is the extent to which it is involved in transepithelial transport of nutrients, ions and water which has not been investigated directly. In *Loligo* limited evidence was presented by Bidder (1950) for some absorption in the intestine while caecal absorption is in progress noting that the intestine may have a more major role when the gonads enlarge, limiting filling of the caecal sac. The capacity to absorb nutrients from contents bypassing the caecum/digestive gland but which have been exposed to digestive enzymes would be an advantage, as would involvement in water and ion homeostasis.

The anal opening in *O. vulgaris* has paired muscular anal flaps or wings. Their absence in species lacking an ink sac suggests involvement in inking rather than a digestive tract function but further investigation is required. The flaps are innervated by the atrio-rectal nerve branch of the visceral nerve and Young (1967, figure 18, p. 9) noted the presence of a plexus of fine fibres in the flap commenting “perhaps afferent.”

The faeces are formed as mucus coated ropes but their composition has not been studied in any detail and the mechanisms, presumed to be neural, controlling of defaecation are unknown (*see* Ponte et al., 2017 for discussion).

### Toxin secretion by the salivary glands: a cornucopia

The toxicity of the exocrine secretions from the posterior salivary glands of *O. vulgaris* was first noted by Lo Bianco working at the Stazione Zoologica Naples, in the early C19^th^ but the toxic fraction was not identified until 1959 (Ghiretti, 1959). Two main constituents were identified with differing biological effects on crabs; amines inducing hyperexcitability and cephalotoxin, lethality. Cephalotoxin is a glycoprotein now known to occur in α and β forms (Cariello and Zanetti, 1977). The current view is that the saliva is a chemical cocktail including tachykinins (i.e. Eledoisin from *Eledone*, Erspamer and Anastasi, 1962), OctTK 1, phospholipase A2, other proteins, and enzymes (e.g., peptidase, hyaluronidase, chitinase) that also play key roles in toxicity of coleoid venom, supplemented by other small organic molecules, peptides, and non-enzymatic proteins (for review *see*: Ruder et al., 2013; Gonçalves and Costa, 2021).

Analysis of the phylogenetic history and molecular evolution of coleoid venom shows comparable diversity and complexity with those found in snakes (Ruder et al., 2013) with evidence of active evolution of some constituents (e.g., cysteine-rich pacifastin and kallikrein). Characterization of the molecular structure and composition of cephalotoxins is still incomplete (maybe except for *Sepia esculenta* cephalotoxin, Ueda et al., 2008). Apart from the glycosylation of the protein, characterization allowed the identification of conserved domains: EGF-like, Sushi, TSP type-1 and LDL-receptor class A (review in Gonçalves and Costa, 2021). Of particular interest is the epidermal growth factor (EGF) domain known to paralyse crustaceans *via* Na_v_ block; cephalotoxins, should be described as EGF neurotoxins. The increasing availability of cephalopod genomes has provided insights about tissue-specific genes expressed in the posterior salivary gland of three cephalopod species (non-tetrodotoxin-bearing octopods vs. tetrodotoxin in *Hapalochlaena maculosa*; Whitelaw et al., 2020).

Although cephalopod salivary gland research is focused on the nature of the toxins and their biological effects little is known about the physiological mechanisms involved in their secretion, how the ratio of the various constituents is determined, and the neural control of secretion together with the transport of saliva along the ducts. The discrete nature of the salivary glands makes them suitable for *in vitro* studies of intact glands and subsequently isolated cells comparable to those applied to mammalian salivary glands.

### Major anatomical differences in the digestive system between species: physiological implications

Knowledge of the physiological consequences of structural similarities and differences in the digestive tract between species (“functional morphology,” Mangold and Young, 1998) is essential to understanding how the animal adapts to its ecological niche. Mangold and Young (1998) and Voss (1988) utilised digestive tract morphology for systematics and revealing phylogenetic relationships. A recent review of the structure and function of the digestive system in molluscs makes more general comparisons (Lobo-da-Cunha, 2019).

#### Crop

The crop is present in Nautiloidea, Vampyromorpha and in most Octopoda but may be reduced or absent in some cirrates (e.g., *Cirrothauma*, Aldred et al., 1983) with Voss (1988) proposing that this is related to a diet of small, soft-bodied prey. Naef (1928) includes a crop in their diagram of a “primitive hypothetical cephalopod.” The crop is absent in Sepiodea and Teuthoidea; molecular techniques should be able to identify the gene(s) responsible. As the primary function of the crop is storage, its absence in Sepiodea and Teuthoidea arguably limits their ability to optimise feeding opportunities unless the stomach is adapted to compensate by being more sacculated and distensible than the stomach in Octopoda.

#### Caecum

A caecum of variable complexity is present in Nautiloidea and Coleoidea but a caecal sac is only present in Teuthoidea and even then, not all Oegopsida (Mangold and Young, 1998) although the functional implications are unknown. The complexity of lamellae in the caecum is less in cuttlefish compared to octopus and squid (Anadón, 2019).

#### Sphincters

In contrast to the poor evidence for structures (sphincters or valves) in the octopus DT there are more detailed descriptions in *L. vulgaris*. Williams (1909) describes a “very remarkable five-way valve” (p. 36) between the caecum and neighbouring cavities of the gut and also sphincters at the openings from the oesophagus and the stomach. Attached to the caecal wall, Bidder (1950) describes a solid valve structure reinforced by cartilage with an innervated “sphincter-like” muscle surrounding its edge and capable of regulating the passage of fluid into, or out of, the caecum at various phases of digestion. Additionally, the sac-like posterior caecum can be separated from the more complex anterior portion (appendix, ciliated organ accessible by the digestive gland and intestine) by a “diaphragm-like” sphincter (Bidder, 1950).

#### Intestine

Regarding the intestine, the principle difference visible externally is the presence of coils or loops (Mangold and Young, 1998) occurring approximately half-way between the junction with the caecum and anal opening. These are present in Sepioidea, Nautiloidea and Octopoda although variably in the Cirrata (e.g., absent in *Cirrothauma*; Aldred et al., 1983) and absent in Teuthoidea and Vampyromorpha. In species with loops and coils the overall intestinal length will be relatively longer in relation to body size but we do not know if the proportions of intestine, rectum and anal regions differs. Loops and coils in the intestine increase the overall luminal surface area for absorption so may indicate differing absorptive capacity. Coiling also increases the relative length of the intestine in relation to body size and potentially the time that digesta spends in the digestive tract, but formal studies supporting this hypothesis are lacking. There have been no comparisons of the coiled area with adjacent regions to investigate structural specialisations and there is insufficient data to identify potential relationships between food type, morphological differences in other structures (e.g., ± crop, ±caecal sac) and intestinal complexity. Internal differences in the intestine have not been reported systematically but transverse septa are present in the proximal intestine of *Nautilus* (Budelmann et al., 1997) but not octopus and the typhlosole is more complex in *Nautilus*.

The above brief descriptions are from a relatively limited number of species but demonstrate that that it would be unwise to extrapolate data on physiology of the digestive tract from one species to another.

## Techniques for the study of digestive tract anatomy relevant to physiology

### Descriptive morbid anatomy

*Post mortem* dissection of fresh specimens is the classical method for describing the gross anatomy. In fresh specimens, it is possible to view and measure the distribution of contents and classify their nature, i.e. liquid, solid and colour (Figure 2); note a description of changes in squid stomach contents with time in Wallace et al. (1981) and Boucher-Rodoni (1976) for caecal contents in *Eledone cirrhosa*. Care should be taken in interpretation as the method of killing may modify digestive tract motility (e.g., brain destruction will stimulate neurones; magnesium chloride relaxes smooth muscle; Bacq, 1934) and contractions may occur *post mortem* redistributing contents, particularly liquids. Guidance on dissection techniques is given in Øresand and Oxby’s (2021) illustrated guide to the dissection of the bobtail squid, *Sepietta oweniana* and by Guerra (2019) for several species.

Useful data can also be obtained from dissection of fixed specimens but distortion can occur and fixation of entire (larger) animals is a challenge. Freezing intact animals immediately *post mortem* preserves the tissue and maintains anatomical relationships.

### Quantitative anatomy

Gross dissection provides largely descriptive, qualitative information on the morphology of the digestive system enabling comparison between species (*see* above). However, quantification of the wet weight of identified tissues provides data enabling further differentiation of species, assessment of the impact of different diets (e.g., natural vs. prepared/synthetic/elaborated) and investigation of potential correlations with environmental changes.

Most DS organ weight data is available for the DG; for example: six decapodiform cephalopods (Omura and Endo, 2016), *O. vulgaris* (García-Garrido et al., 2010; Bastos et al., 2020), *Octopus maya* and *Octopus mimus* (Linares et al., 2015; Pascual et al., 2019) with more limited data for the stomach and caecum (e.g., Omura and Endo, 2016). Detailed analysis of digestive gland weights has shown changes in the weight of the digestive gland in O. *maya* with season and location (Pascual et al., 2019).

Ideally, data on dry weight should also be obtained to differentiate between increase tissue mass compared to increased water content which may be pathophysiological (e.g., oedema).

### Imaging techniques

Dissection is a destructive process so should only be used when there are no other methods available. For example, high-field magnetic resonance imaging and micro-computed tomography have been used for external and internal taxonomic description of a preserved specimen of a novel species of dumbo octopus (*Grimpoteuthis imperator*, Ziegler and Sagorny, 2021). The resolution of the digestive system was sufficient to identify all key features (e.g., presence of crop and “pancreatic” portion of the DG, paired hepatic ducts) and also to note that there was only a single posterior salivary gland and the anal flaps were absent (as was the ink sac). Importantly, collection of this type of imaging data enables construction of 3D models of the viscera so that the relationships of structures can be readily examined. High resolution ultrasound can also be used to image tissue in preserved animals but it has particular utility for anatomical and physiological studies in living animals so is discussed below.

### Histology, histochemistry and immunohistochemistry

The following are *selected examples* to illustrate the potential contribution of histological techniques to understanding physiology.

#### Muscle layers

The muscle layers and the presence of connective tissue has mainly been investigated using haematoxylin and eosin (H&E) and Masson’s trichrome (e.g., Anadón, 2019) with a few studies using transmission electron microscopy (reviewed by Budelmann et al., 1997). Histology enables identification of the muscle type (e.g., smooth or striated), the orientation (e.g., circular, longitudinal, oblique) and thickness of the layers in the various regions. This knowledge has functional implications as smooth and striated muscles have different physiological properties. When the muscle is relatively thin it is suggestive of areas of storage and when thicker, indicative of more powerful contractions or sphincter areas. As noted above, evidence for the presence of sphincters in the digestive tract is limited and is an area where histological studies can inform understanding of control processes. The complex valves involving both muscle and mucosal modifications reported in squid (Williams, 1909; Bidder, 1950) require histological investigation particularly to investigate the claim (Bidder, 1950, p. 18) that they are “reinforced by cartilage.”

The basic organization of the muscle in the cephalopod digestive tract is an inner layer of longitudinal muscle and an outer layer of circular muscle with some regions having an additional outer longitudinal layer (e.g., *O. vulgaris* oesophagus, Fernández-Gago et al., 2019) or a circular layer mixed with oblique muscles (e.g., thick stomach muscle in *E. megalocyathus*, Garri and Lauria de Cidre, 2013). Significantly, this contrasts with the vertebrate digestive tract where broadly the organisation is an inner circular muscle layer and an outer longitudinal one (Furness, 2006); the functional consequences particularly for the mechanisms underlying peristalsis require investigation.

#### Epithelial secretions

Using H&E, Fernandez-Gago et al. (2019, p. 324, table 1) surveyed the epithelial cells secreting mucus or a granular secretion throughout the digestive tract of *O. vulgaris* and similar data is available for *E. megalocyathus* (Garri and Lauria de Cidre, 2013). Mucus secretions were classified using alcian blue (acidic/sulphated/carboxylated glycoconjugates) and periodic acid Schiff (neutral glycoconjugates) alone or in combination. Of particular physiological relevance is the differential distribution of the various types of mucus secreting cells in the lamellae and first and second coils of the caecum (Fernández-Gago et al., 2019) in *O. vulgaris*.

Further insights into secretions are gained from studies of ultrastructure (e.g., electron dense granules, rough endoplasmic reticulum) and enzyme immunohistochemistry (Westermann and Schipp, 1998a). In *N. pompilius* digestive tract the most prominent enzymes were acid and alkaline phosphatase and β-glucuronidase, the latter implicated in mucus catabolism but chymotrypsin and trypsin-like enzymes implicated in nutrient digestion were also present (Westermann and Schipp, 1998a).

#### Morphological changes related to function

In the DG marked quantifiable histological changes occur during the course of digestion. DGs showing large numbers of “boules” are considered to be active as these contain digestive enzymes ready for exocytosis (Bidder, 1957). The number of “boules” decreases with time after feeding and are absent in one to 3 days without food in *O. vulgaris* (Bidder, 1957). Brown and grey bodies associated with the excretion of waste products are seen during the excretory phase (Bidder, 1957; Rodrigo and Costa, 2017).

#### Neurones and neurotransmitters

The innervation of the digestive tract in cephalopods has been relatively neglected since the detailed anatomical and histological studies by, for example, Alexandrowicz (1928), Bogoraze and Cazal (1946) and Young (1967; 1971) with recent studies only commenting in passing on the presence of neurones (e.g., Anadón, 2019; Fernández-Gago et al., 2019). Here we use studies of the gastric ganglion to exemplify the contribution of histological techniques. The gastric ganglion is a prominent oval structure (∼3 mm long in a 500 g – body weight - *O. vulgaris*) located on the external surface of the digestive tract at the junction of the stomach, caecum and intestine (Figure 2). It is composed of a cortical layer of cells with an inner neuropil from which nerve fibres emerge to supply all adjacent regions of the DT including the digestive gland ducts. The neuronal organisation of the ganglion has been investigated using cresyl violet (Andrews and Tansey, 1983a), Picro-Ponceau (Baldascino et al., 2017), Golgi-Cox (Spasiano, 2020) various modifications of Cajal’s silver stain methods (Young, 1971) and antibodies for neurofilament 200, neuronal marker acetylated alpha-tubulin and neuronal nuclear antigen (NeuN, Baldascino et al., 2017). The overall organisation of the cells and the connectivity of axons and dendrites closely resembles the organisation of the central nervous system, supporting the view that it is a major peripheral control centre for the digestive tract. Conventional, fluorescence and immuno-histochemistry (including for the presence of synthetic or destructive enzymes) have identified the presence (usually by “like-immunoreactivity”) of potential neurotransmitters including acetylcholine, corticotrophin-releasing factor, dopamine, FMRF-amide, gamma amino butyric acid, 5-hydroxytryptamine, nor-adrenaline, octopamine (*see* Baldascino et al., 2017 for methodological details and references); the functions of these substances now need investigating using the physiological techniques discussed below. Although we have focused on the tract itself there is evidence that the vasculature supplying the tract is innervated (Andrews and Tansey, 1983b) which is of importance in matching blood flow changes to the post prandial metabolic demands.

## Methods used for the investigation of major physiological functions of the cephalopod digestive system

The majority of the *in vivo* techniques will be regulated by national legislation (e.g., Directive 2010/63/EU) and/or institutional review. Collection of animals from the wild may also require specific authorisation. Removal of digestive tract tissue for study *in vitro* requires a humane method of killing (*see* Andrews et al., 2013; Fiorito et al., 2015) and although the EU legislation does not currently specify methods for cephalopods, unlike the situation for vertebrates, in Annexe IV the principles to be followed are specified. For all studies involving animals, researchers should carefully consider application of the 3Rs (Fiorito et al., 2014), experimental design, for example by using the Experimental Design Assistant, and consult animal experimentation reporting guidelines such as ARRIVE (Kilkenny et al., 2010; Percie du Sert et al., 2020). Some research on the cephalopod digestive system is of direct relevance to aquaculture so note should be taken of the growing concerns regarding welfare (Jacquet et al., 2019; Birch et al., 2021).

### Measuring motility of the digestive tract

Motility describes the way the contraction and relaxation of the muscles of the digestive tract move the secretions (including salivary and DG ducts), the food whilst it is digested and absorbed, and any indigestible components, waste products, adherent mucus and shed epithelial cells.

#### Whole digestive tract and regional transit time

Whole DT transit time is usually taken as the time from when food enters the gut to when the undigested constituents of that meal leave the DT as faeces but it can also be applied to the time taken for an artificial marker to pass through the DT. Although conceptually it is a simple measurement, the value obtained depends upon the physical and chemical nature of the food, the physiology of the DT which may be influenced by external stressors, ambient temperature in poikilothermic species and any conscious control over motility such as deferral of defaecation. Although whole digestive tract transit time provides some information about the physiology of digestion it gives little insight into each region’s contribution to the overall time and hence its function (e.g., how long do different foods spend in the stomach?). Measurements of overall and regional times are required when investigating control of the digestive tract as well as quantifying the impact of different diets and environmental changes (e.g., temperature). Two main methods have been used.

##### Movement of food labelled with markers

The movement of shrimps (*Crangon crangon*) labelled with barium sulphate was monitored in juvenile *N. pompilius* by taking timed X-ray photographs (Westermann et al., 2002). Although this technique allows non-invasive monitoring of food movement once the shrimps lose their integrity the barium will disperse into the liquid contents of the tract so may move at a different rate from the solid components. Indigestible food entered the rectum 4 h after feeding but spent a further 8 h in the rectum before defaecation; the overall duration of digestion is assessed at 12 h (at 18–19°C). This technique has not been used in other cephalopods but is applicable (particularly to cuttlefish) provided humane methods of restraint during imaging can be developed. X-rays can also be used to monitor the passage of solid radio-opaque markers of different size incorporated into the food.

##### Food distribution in the digestive tract

This usually involves feeding matched (e.g., weight, sex) groups of animals a known amount of food after a period of food deprivation, e.g., 24 h for *O. vulgaris* or *S. officinalis* (*see* Sykes et al., 2017 for assessment of welfare impact), killing groups at fixed times after feeding and measuring the amount and composition (e.g., pH, enzyme activity, lipid and protein content) of contents in the lumen of each region of the tract; some studies also include biochemical analysis of the digestive gland and haemolymph sampling. The change in the amount and composition of digesta in each region of the gut (and potentially the faeces) can be used to assess the overall time taken to digest food and the contribution of each region. This approach has been used in *Octopus maya*, *O. mimus* and *O. vulgaris* type II from Brazil (Martínez et al., 2012; Linares et al., 2015; Gallardo et al., 2017; Bastos et al., 2020).

Using standardised food intake this method can also be used to estimate the rate at which food leaves the stomach as shown in cuttlefish (Quintela and Andrade, 2002a; b). With a shrimp meal gastric emptying in *S. officinalis* was shown to be faster at higher ambient temperatures; the time for 50% of the meal to be emptied from the stomach was estimated to be 3.5 h at 15.5°C, but 1.6 h at 23°C (Quintela and Andrade, 2002a) but this method does not allow the relative contributions of the effects of higher temperature on enzyme activity and motility to be identified. Techniques need to be developed to measure the emptying of the solid and liquid components separately as liquid components are likely to empty more quickly; in cephalopods this may be important as in octopus soluble nutrients enter the digestive gland and haemolymph ∼ 40 min post ingestion (Rosa et al., 2004; Linares et al., 2015). This food distribution method is readlily applicable to meaure emptying from the stomach in cuttlefish and squid where there is no crop, but in octopus where the crop and stomach act in concert other techniques will need to be developed to measure exchange between the two regions as well as net emptying into the caecum/intestine. Finally, a key reason why measurement of gastric emptying is needed is to understand how the process is regulated to ensure that only suitable processed food enters the caecum and the relationship between gastric emptying and satiety/hunger signals.

The main disadvantages of this technique are that it requires the death of the animal, uses a large number of animals depending on the temporal resolution studied, groups need careful matching so data can be combined to produce a single plot of the timing of various processes, contents may move after death because of the interconnectivity of the gut regions and *post mortem* continuation of motility. However, currently it is the only technique allowing sampling of gut contents (except faeces) enabling detailed biochemical analysis to follow the course of food digestion and also the analysis of exocrine secretions contributing to digestion. If an animal is killed, we urge that optimal use is made of the all the tissues by making them available to other local researchers or making a bank of fixed or frozen tissue; the latter is particularly important for rarer species.

#### *In vitro* studies of physiology and pharmacology

*In vitro* here refers to studies in which either the entire digestive tract, a region (e.g., the crop, the rectum) or a strip of tissue cut from a region is removed from an animal killed humanely is placed in a tissue bath. Functionality is maintained by immersion in a physiological solution (modified Ringer’s solution), gassed (to maintain oxygen and the pH of any buffers) and kept within normal body temperature range while measurements are made of muscle contractile activity (e.g., tension or intra-luminal pressure). Here we focus on techniques appliable to either segments of digestive tract or the entire digestive tract but do not discuss *In vitro* techniques applied to studying the working of the buccal mass (Boyle et al., 1979).

With appropriate experimental design and controls the techniques described below can be used to investigate the effects of a range of interventions and conditions applied before the *in vitro* study but which are likely to have prolonged effects. Examples include: *1*) Life stage dependent changes; *2*) Effect of protracted exposure to expected changes in environmental conditions associated with climate change (e.g., sea water acidification); *3*) Comparison of diets with differing composition, energy density and digestibility; *4*) Investigation of functional effects of endogenous agents and/or their receptors which may be identified from genomic studies (e.g., cephalopod G-protein receptors, Ritschard et al., 2019), neuropeptidome analysis (e.g., Zatylny-Gaudin et al., 2016), molecular and immunohistochemical analysis of the gastric ganglion (e.g., Baldascino et al., 2017).

##### Tension measurement

*In vitro* recordings of tension from various regions of the digestive tract have been undertaken in *O. vulgaris* (e.g., Andrews and Tansey, 1983a; Takuwa-Kuroda et al., 2003), *S. officinalis* (e.g., Bacq, 1934; Zatylny-Gaudin et al., 2010) and *Doryteuthis pealeii* (e.g., Wood, 1969). However, the paucity of studies shows that this is an under-utilized technique in cephalopods. It is also possible to measure pressure in closed isolated segments of digestive tract (Andrews and Ponte, personal observations).

Two insights into the physiology of digestive tract motility emerge from published studies:

###### Spontaneous contractile activity

**FIGURE 3:**
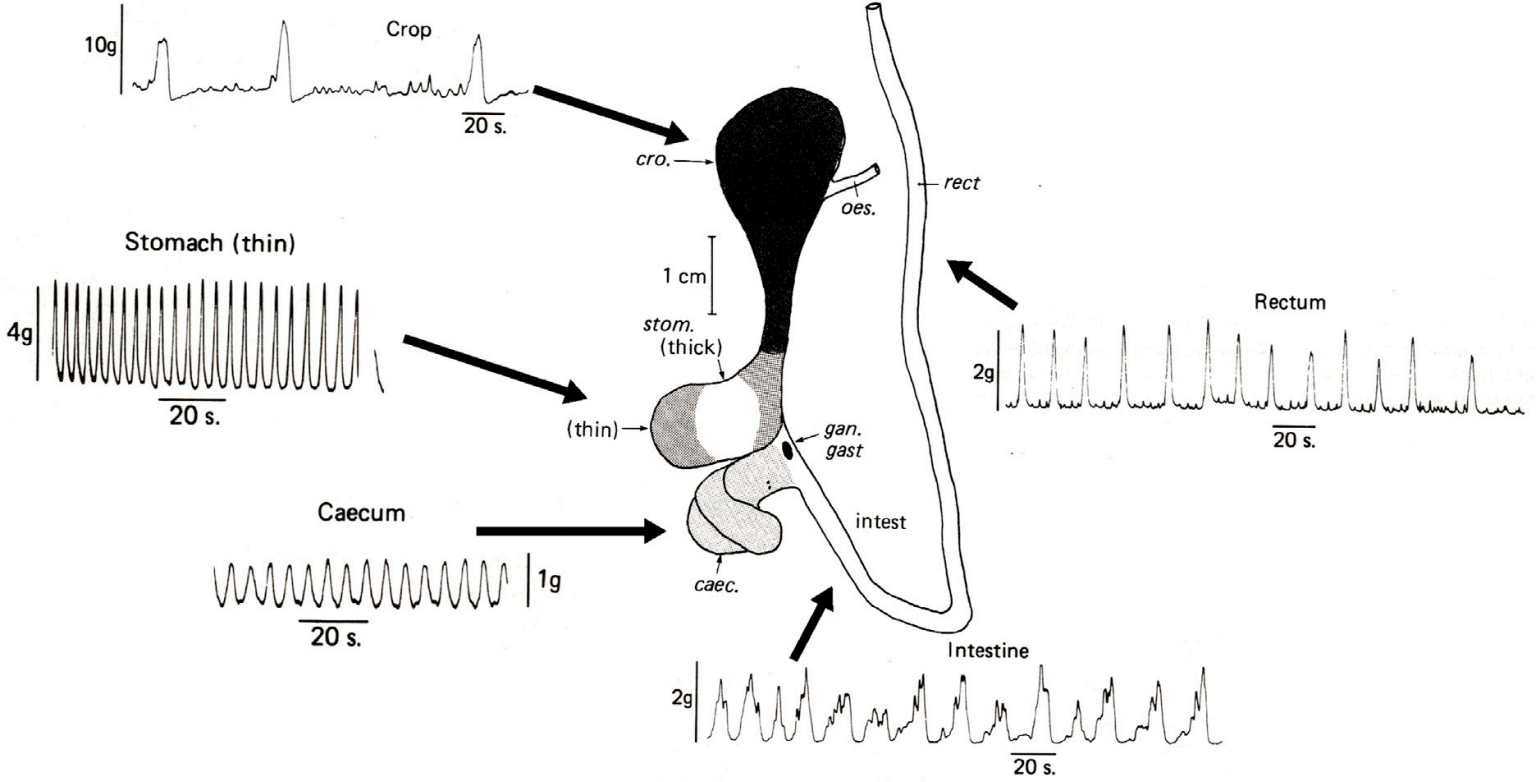
Spontaneous contractile activity recorded *in vitro* from longitudinal muscle strips of the main regions of the digestive tract removed immediately *post mortem* from *Octopus vulgaris*. Note that all regions show some spontaneous contractile activity but that the magnitude and frequency differs. Abbreviations: caec = caecum; cro = crop; gan. gast = gastric ganglion; intest = intestine; rect = rectum. Modified from Figures 1 and 4 in Andrews and Tansey, 1983a, pages 111 and 115. Reproduced with the application of an author permissions waiver request from Cambridge University Press.

All regions of the digestive tract show varying degrees of spontaneous (i.e., in the absence of overt stimuli) contractile activity with contractions of differing amplitudes and frequencies (Figure 3). Myogenic activity (i.e. not requiring neurones) is particularly important to investigate as in vertebrates, including fish (e.g., *Myoxocephalus scorpius* Brijs et al., 2017b) and humans (Hwang et al., 2009), this is due to activity of the Interstitial Cells of Cajal (ICC) which act as pacemaker cells periodically depolarizing to initiate contraction of adjacent smooth muscle cells (Sanders, 2019). Wood (1969) reported that contractile response of the squid stomach to stretch continued in the presence of tetrodotoxin as did the associated electrical activity providing very preliminary evidence for “myogenic activity.” Functional studies, combined with molecular and immunohistochemical studies (e.g., calcium activated chloride channel ANO-1 positive cells) to investigate the presence of ICCs are required to identify whether the fundamental mechanism (ICC-smooth muscle cell) underlying digestive tract contractile activity in vertebrates occurs in cephalopods.

###### Modulation by a diversity of putative neurotransmitters and hormones

**FIGURE 4:**
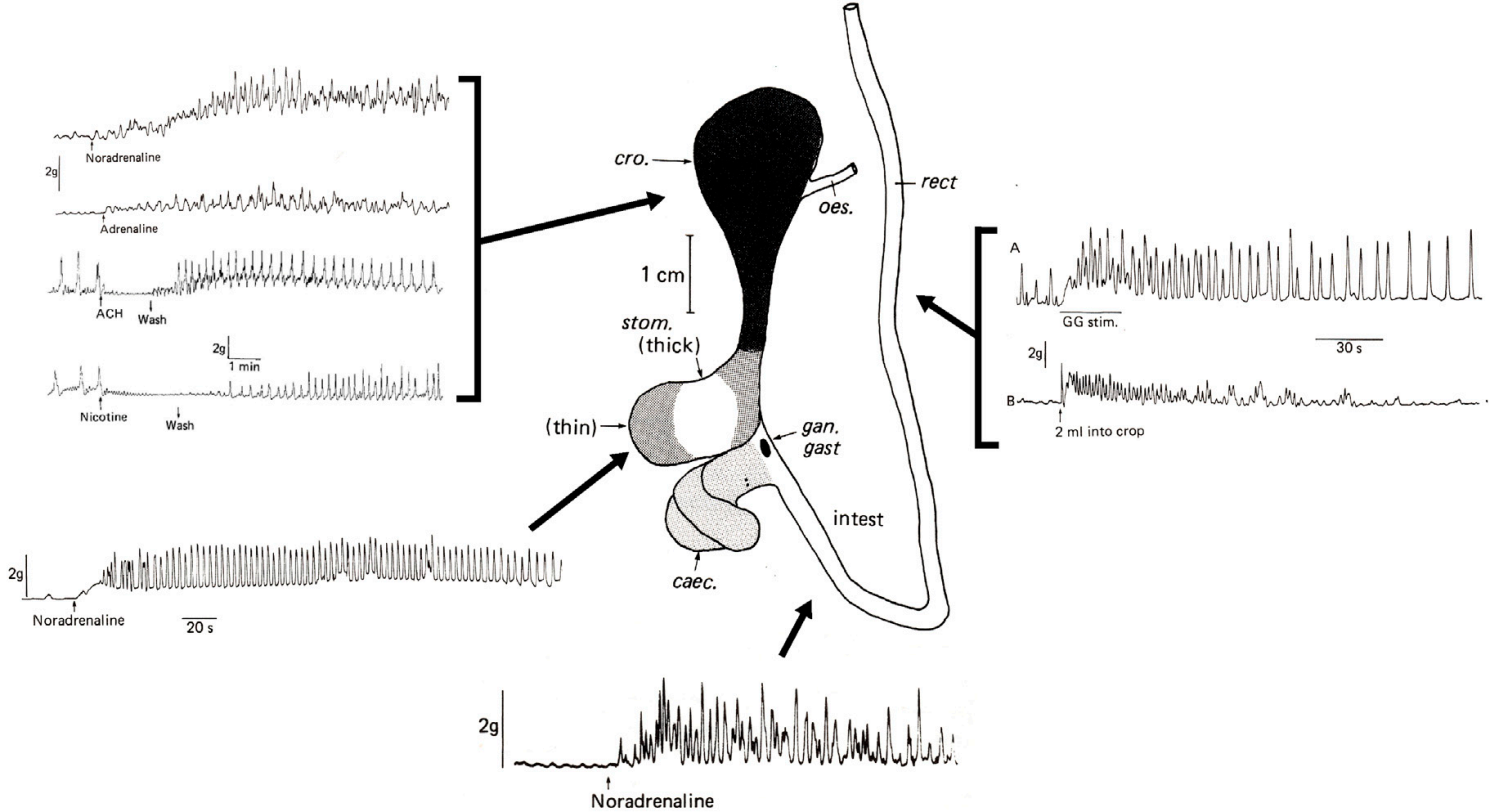
The effects of adreno- and cholino-receptor ligands on the *in vitro* contractile activity of main regions of the *Octopus vulgaris* digestive tract. The crop, stomach and intestine all show an increase in contraction amplitude and tone to noradrenaline (1 µg/ml–10 μg/ml) with the response to adrenaline (2 μg/ml) in the crop also shown. Both acetylcholine (ACH; 20 μg/ml) and nicotine (20 μg/ml) cause transient inhibition of ongoing contractile activity in the crop followed by rebound excitation following washing. The upper right -hand panel shows the excitatory effect of gastric ganglion (GG) stimulation (20 V, 0.5 ms, 20 Hz) on the crop and the stimulation of crop motility by fluid distension (2 ml); these two recordings are from *in situ* preparations. Abbreviations: caec, caecum; cro, crop; gan. gast, gastric ganglion; intest, intestine; rect, rectum. Details of methods are in Andrews and Tansey, 1983a. This Figure is modified from Figures 1, 8, 9 and 10 in Andrews and Tansey, 1983a, pages 111, 119, 121 and 122. Reproduced with the application of an author permissions waiver request from Cambridge University Press.

Figure 4 shows a stimulatory effect (increased tone, contraction amplitude and frequency) of nor-adrenaline/adrenaline on the crop, thin part of the stomach and intestine while acetylcholine and nicotine inhibited activity which recovered above baseline levels on washing (Andrews and Tansey, 1983a). This provides preliminary evidence for reciprocal control of motility analogous to divisions of the autonomic nervous system in vertebrates (Olsson, 2009). An inhibitory effect of acetylcholine followed by post-washing excitation has also been reported in squid stomach as has the excitatory effect of adrenaline (Bacq, 1934; Wood, 1969). Although responses to the cholinergic and adrenergic receptor agonists are clear, defining receptor types/subtypes involved requires the use of selective or specific receptor antagonists (*see* IUPHRA/BPS Guide to Pharmacology; www.guidetophamacology.org). Whilst many receptors are relatively well characterized in mammalian tissues very little information is available regarding the pharmacology of receptors in cephalopods defined either by agonists or antagonists.

###### Imaging of contractions: spatiotemporal mapping

The external profile of the digestive tract changes with the progression of contractions and also as the degree of distension of a particular segment is changed by bolus progression. High resolution video recording of the digestive tract followed by automated computer analysis of the apparent diameter enables the generation of “spatiotemporal maps” showing the contractile activity, its magnitude, direction (oro-anal or ano-oral) and speed of travel (*see* Lentle and Hulls, 2018 for review of the method). Spatiotemporal mapping frequently used in mammals to investigate the effects of drugs (e.g., in mouse stomach, Worth et al., 2015) has also been utilised to study motility in the digestive tract of the fish *Oncorhynchus mykiss* (Brijs et al., 2017a) and *Myoxocephalus scorpius* (Brijs et al., 2014; Brijs et al., 2017b). However, it has not been applied to cephalopods, although using cursor measurement of individual video frames at timed intervals Sykes et al. (2020; Figure 3B) demonstrated its applicability to analysis of crop contractions using an *in vitro* preparation in *O. vulgaris*.

Other techniques also applicable *in vitro* to investigate the effect of treatments *in vivo* (e.g., acute food deprivation, adaptation to different diets, pharmacological or surgical interventions to investigate control) on motility measured *in vitro* include the passage of artificial boluses (e.g., Le et al., 2021), electrophysiological studies of muscle using extracellular (e.g., suction electrodes as in squid stomach, Wood, 1969) and intracellular (adapting techniques used in octopus arm muscle, Rokni and Hochner, 2002) recordings.

#### *In vivo* imaging

Imaging the digestive tract *in situ* in the living animal (*in vivo*) allows measurement of the movement of food, changes in the physical nature of the contents through the tract and characterisation of the contractile activity. However, methods used currently require some form of restraint or possibly sedation which may themselves affect motility, although in some species it may be possible to train the animal to remain quiescent during monitoring. Whilst X-rays have been used to monitor the progress of food mixed with radio-opaque barium sulphate in *N. pompilius* (Westermann et al., 2002) and *S. officinalis* (Ponte et al., 2017, *see* also Figure 1H therein) the low temporal resolution does not provide information about individual contractions. Additionally, movement of the contrast medium may not accurately reflect the bolus movement.

The technique most applicable to cephalopods is the use of high-resolution ultrasound (e.g., VEVO - Visualsonics). In a pilot study of unrestrained *O. vulgaris*, Ponte et al. (2017) were able to show propulsive contractions of the crop in both longitudinal and transverse planes, and constriction of the caecum together with the motion of the complex spirally organised lamellae (*see* figure 1 in Ponte et al., 2017). The method also provides information about the physical characteristics of the lumen contents. This technique should be adaptable to cuttlefish, although the locomotor activity of squid may prevent use without undue restraint. Combination of *in vivo* ultrasound with the *in vitro* techniques outlined above will identify if motility is modified by diets of different composition, and the impact of environmental changes.

The transparency of *O. vulgaris* paralarvae post-hatching, enables direct observation of the digestive tract during feeding allowing observation of the distribution of food in the digestive tract. Analysis of high-resolution video recordings enabled quantification of crop volume, contraction frequency in crop, stomach and intestine, and insights into the overall timing of digestion (Nande et al., 2017). Juvenile and mature individuals of several species of cephalopod are sufficiently transparent for direct observation of the passage of material through parts of the digestive tract and also to see changes in the colour of the digestive gland. Bidder (1950) utilised the transparency of juvenile squid to make observations on the movement ingested pieces of fish (marked with iron saccharate, carmine and Nile blue) in the digestive tract commenting that “the only parts of the digestive system not visible in the living animal are the buccal glands and poison glands and the “pancreas” (Bidder, 1950, p. 8). Direct observation is not an option for cuttlefish species because of the cuttlebone but there are several examples of transparent/semi-transparent squid (e.g., Cranchiide family) and octopus (e.g., Vitreledonellidae family).

### Measuring food ingestion rate

Ingestion rate can be measured in experimental settings using live or frozen organisms, pieces of natural or processed (elaborated/synthetic) food and is calculated as the difference between the delivered food and that remaining for at least 2 h after being offered to the animals. The latter time can be established according to the feeding time regularly used in the laboratory depending on the species feeding habits. The percentage of leached nutrients must be measured and depends on the type of the diet. When processed food is used, leaching is measured using the shaking method (Obaldo et al., 2002). For this, 10 samples of 2 g of each diet are placed in 250-ml flasks placed in a horizontal shaker for 2 h (or the time that is estimated in each experimental condition to feed the animals) at the same temperature and water quality used experimentally. After that time, the water is filtered (pre-weighed, Whatman 1441-090 is recommended) to separate the remaining food from the leached water. Original food (*N* = 10) and leached samples of each diet are dried in a convection oven at 60°C for 48 h until constant weight, and then cooled in a desiccator. Dried feed samples are weighed and analysed for dry matter retention. Ingestion rate is calculated according to the following equation:

Ingestion rate(I)=[offered food-recovered food]×[1−(nutrient leaching)]

where offered and recovered food are expressed as dry weight (g) (60°C, 48 h), and unconsumed food as % of the nutrients lost during the shaking procedure (Obaldo et al., 2002). Ingestion rate (I) is expressed as g. ingested food (wet weight) considering the water content obtained after dry sampling at 60°C for 48 h. In the case of fresh or frozen food the same method is used. A review of the stomach contents and ingestion rate in cephalopods by Ibáñez et al. (Ibáñez et al., 2021) gives recommendations for obtaining data of ecological importance.

### Measuring the time course of digestion

Digestion time differs between species, with temperature, dissolved oxygen and type of food, among other variables. For that reason, when evaluating the digestive processes, preliminary observations are required to determine the overall duration of digestion and hence sampling frequency. In *O. vulgaris sensu stricto*, *O. vulgaris* type II from Brazil, *O. maya*, and *O. mimus* fed fresh crab at 5% of the body weight it took ∼ 400 min to complete the digestion process (Martínez et al., 2012; Linares et al., 2015; Gallardo et al., 2017; Bastos et al., 2020). Depending on the number of animals available, it is possible to take timed samples during the process, but how many animals should be sampled to maintain a balance between 3Rs considerations and data quality? The answer depends on the type of analysis required, but usually a minimum of three animals will be necessary to obtain data that can be statistically analysed (Table 2). Also, it is essential to remember that digestive juice enzyme activity can be highly variable, especially with a mixed diet. For that reason, in this type of study it is highly recommended to use a single type of food to evaluate digestion timing (crustacea are arguably the best diet because their importance in trophic ecology of cephalopods).

**TABLE 2:** Number of animals sampled in three octopus species when the timing of the digestive process was studied.

*O. maya* ^a^		*O. mimus* ^a^		*O. vulgaris* type II^b^	
min	*N*	min	*N*	min	*N*
0	6	0	5	0	3
20	7	30	3	60	4
40	7	90	3	120	5
80	7	150	3	200	4
120	7	210	3	300	4
180	8	270	3	400	4
240	10	330	3		
360	6	390	3		
480	3				

Note that animals were fed with one piece of crab (*Callinectes* spp for *O. maya* and *O. vulgaris* type II and *Cancer* spp for *O. mimus*) except for animals at time zero. All the animals should be fasted at least by 12 h before the experiment. After fasting, allow the octopus to ingest the crab for enough time to guarantee that animals end the ingestion process; inn *O. maya, O. mimus* and *O. vulgaris* type II this takes around 20–30 min.

a Linares et al. (2015).

b Bastos et al. (2020).

Table 3 shows the variety of digestive enzymes in octopus species. Although there are probably more than a dozen types of enzyme with different roles, there is evidence suggesting that the acidic enzymes have higher activity during digestion (e.g., Ibarra-García et al., 2018). Based on Boucaud-Camou and Boucher-Rodoni (1983), Martínez et al. (2012) studied the pH of the “gastric juice” (i.e., the fluid found in the stomach but not secreted by the stomach) of *O. maya* during digestion of crab. They found that pH varied between 5.2 and 6, demonstrating, as was previously observed in *O. sinensis* (?) by Morisita (1972 a, b and c, cited by Boucaud-Camou and Boucher-Rodoni, 1983) that the main enzymes in the digestive tract of those octopus species were acidic proteinases. Until now, besides *O. sinensis*(?) and *O. vulgaris* sp. (Table 3) the presence of acidic proteinases has been demonstrated in the gastric juice of *O. mimus, O. maya* (Linares et al., 2015) and *O. vulgaris* type II (Bastos et al., 2020), and in the digestive gland of *O. bimaculoides* (Ibarra-García et al., 2018), *Robsonella fontaniana* (Pereda et al., 2009) and *Enteroctopus megalociathus* (Farías et al., 2010) indicating that the acidic proteinases may be the main type of digestive enzyme in octopus species. It is interesting to note that in some studies high activities of amylase are reported suggesting that some species *(O. bimaculoides; O. vulgaris sensu stricto)* may have the ability to digest complex carbohydrates (Table 3). Although interesting, these studies require duplication (Table 3) and the sources of complex carbohydrate identified.

**TABLE 3:** Enzyme activities detected with different methods in digestive gland (DG), anterior (ASG), and posterior (PSG) salivary gland, gastric juice (GJ) or entire paralarvae (All) of several octopus species.

Enzymes	Activity	Tissue/organ	Species	Stage	Reference
Acid phosphatases	xx	DG	*E. megalocyathus*	J	Farías et al. (2010)
Acid phosphatases	xxx	All	*R. fontaniana*	Pl	Pereda et al. (2009)
Acid proteinases	xxxx	GJ,DG	*O. vulgaris* Type II	PA	Bastos et al. (2020)
Acid proteinases	x	GJ,DG	*O. vulgaris* Type II	PA	Bastos et al. (2021)
Acidic proteases	xxx	DG	*O. vulgaris* s.e	?	Arvy (1960)
Acidic proteases	xxx	GJ	*O. vulgaris* s.e	?	Boucaud-Camou and Boucher-Rodoni (1983)
Alkaline phosphatase	xx	All	*R. fontaniana*	Pl	Pereda et al. (2009)
Alkaline proteases	xxx	PSG	*O. vulgaris* s.e	?	Arvy (1960)
Alkaline proteinases	x	GJ, DG	*O. vulgaris* Type II	PA	Bastos et al. (2020)
Alkaline proteinases	xx	DG	*E. megalocyathus*	J	Martínez-Montaño et al. (2018)
Amylase	xx	DG	*O. bimaculoides*	J	Ibarra-García et al. (2018)
Amylase	xxx	GJ	*O. bimaculoides*	J	Ibarra-García et al. (2018)
Amylase	nd	SG	*O. bimaculoides*	J	Ibarra-García et al. (2018)
Amylase	xx	PSG, DG, Ca	*O. vulgaris* s.e	A	Mancuso et al. (2014)
Carboxypeptidase A	x	PSG	*O. sinensis (?)*	?	Morishita 1974 a,b,c cited by Boucaud-Camou and Boucher-Rodoni (1983)
Carboxypeptidase A	x	DG, GJ	*O. sinensis* (?)	?	Morishita 1974 a,b,c cited by Boucaud-Camou and Boucher-Rodoni (1983)
Carboxypeptidase B	x	DG, PSG	*O. vulgaris* s.e	?	Mancuso et al. (2014)
Cathepsin B	xxxx	DG	*O. maya*	PA	Rosas et al., Unpublished data
Cathepsin D	xxxx	DG, GJ	*O. maya*	J	Martínez et al. (2011)
Cathepsin H	xxxx	DG	*O. maya*	PA	Rosas et al., Unpublished data
Cathepsin L	xxxx	DG	*O. maya*	PA	Rosas et al., Unpublished data
Cathepsin-like	xxxx	DG, GJ, PSG	*O. sinensis* (?)	?	Morishita 1974 a,b,c cited by Boucaud-Camou and Boucher-Rodoni (1983)
Cellulase	xxx	DG	*O. vulgaris* s.e	?	Boucaud-Camou and Boucher-Rodoni (1983)
Chitinase	xx	DG, St	*O. sinensis* (?*)*	?	Okutani and Kimata (1964)
Chitinase	xx	PSG	*Eledone cirrhosa*	A	Grisley and Boyle (1990)
Chymotrypsin	xx	PSG	*O. sinensis* (?)	?	Morishita 1974 a,b,c cited by Boucaud-Camou and Boucher-Rodoni (1983)
Chymotrypsin	x	All	*O. vulgaris* s.e	Pl	Villanueva et al. (2002)
Chymotrypsin	xxx	PSG,DG,Ca	*O. vulgaris* s.e	A	Mancuso et al. (2014)
Chymotrypsin	xx	GJ	*O. sinensis* (?)	?	Morishita 1974 a,b,c cited by Boucaud-Camou and Boucher-Rodoni (1983)
Chymotrypsin	xx	DG	*Eledone spp*	PA	Boucher-Rodoni (1982)
Chymotrypsin	xxx	PSG	*Eledone cirrhosa*	A	Grisley and Boyle (1987)
Chymotrypsin	xx	DG, GJ	*O. maya*	J	Martínez et al. (2011)
Chymotrypsin	xxx	GJ,DG	*O. vulgaris* Type II	PA	Bastos et al. (2021)
Chymotrypsin	xx	DG	*E. megalocyathus*	J	Farías et al. (2010)
Chymotrypsin	x	DG	*E. megalocyathus*	J	Martínez-Montaño et al. (2018)
Chymotrypsin	xx	DG	*O. bimaculoides*	J	Ibarra-García et al. (2018)
Chymotrypsin	x	GJ	*O. bimaculoides*	J	Ibarra-García et al. (2018)
Chymotrypsin	x	SG	*O. bimaculoides*	J	Ibarra-García et al. (2018)
Dipeptidase	xx	ASG	*O. sinensis* (?*)*	?	Morishita 1974 a,b,c cited by Boucaud-Camou and Boucher-Rodoni (1983)
D-aspartate oxidase	xx	DG	*O. vulgaris*	?	Tedeschi et al. (1994)
Esterases	xxx	PSG	*O. vulgaris* s.e	?	Arvy (1960)
Glucosaminidase	x	All	*R. fontaniana*	Pl	Pereda et al. (2009)
Leucine amino peptidase	x	DG	*O. maya*	PA	Aguila et al. (2007)
Leucine amino peptidase	x	DG	*E. megalocyathus*	J	Martínez-Montaño et al. (2018)
Lipase	xx	DG	*O. vulgaris* s.e	?	Fernández-Gago et al. (2019)
Lipase	xx	DG	*O. vulgaris* s.e	?	Mancuso et al. (2014)
Lipase	xxx	DG	*E. megalocyathus*	J	Martínez-Montaño et al. (2018)
Lipase	xx	DG	*O. bimaculoides*	J	Ibarra-García et al. (2018)
Lipase	x	GJ	*O. bimaculoides*	J	Ibarra-García et al. (2018)
Lipase	xxx	SG	*O. bimaculoides*	J	Ibarra-García et al. (2018)
Trypsin	x	PSG	*O. sinensis* (?)	?	Morishita 1974 a,b,c cited by Boucaud-Camou and Boucher-Rodoni (1983)
Trypsin	x	DG	*O. maya*	PA	Aguila et al. (2007)
Trypsin	x	DG, GJ	*O. maya*	J	Martínez et al. (2011)
Trypsin	x	All	*O. vulgaris* s.e	Pl	Villanueva et al. (2002)
Trypsin	x	All	*O. vulgaris* s.e	Pl	Morote et al. (2011)
Trypsin	xxx	PSG, DG,Ca	*O. vulgaris* s.e	A	Mancuso et al. (2014)
Trypsin	xxx	GJ,DG	*O. vulgaris* Type II	PA	Bastos et al. (2021)
Trypsin	x	DG	*E. megalocyathus*	J	Farías et al. (2010)
Trypsin	x	DG	*E. megalocyathus*	J	Martínez-Montaño et al. (2018)
Trypsin	xx	DG	*O. bimaculoides*	J	Ibarra-García et al. (2018)
Trypsin	x	GJ	*O. bimaculoides*	J	Ibarra-García et al. (2018)
Trypsin	x	SG	*O. bimaculoides*	J	Ibarra-García et al. (2018)
α-amylase	xxx	GJ,DG	*O. vulgaris* Type II	PA	Bastos et al. (2021)
α-amylase	x	DG	*E. megalocyathus*	J	Martínez-Montaño et al. (2018)
β-Galactosidase	xx	All	*R. fontaniana*	Pl	Pereda et al. (2009)

Relative magnitude of the enzyme activity reported by authors (x). When the stage of life cycle was not identified a symbol (?) was added. Pl, paralarvae; J, juveniles; PA, pre-adults.

**FIGURE 5:**
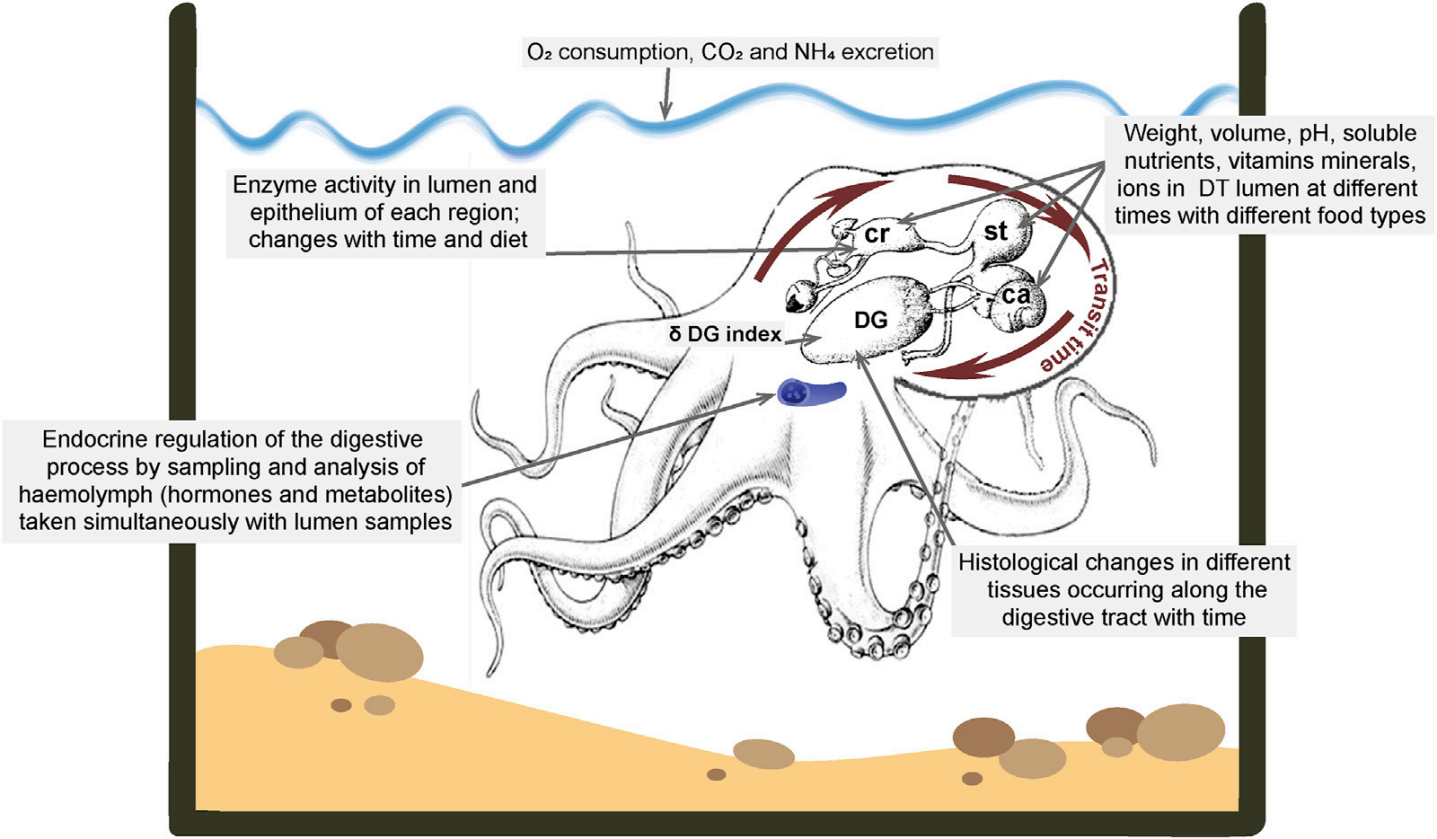
Summary of measurements which can be made in octopus to quantify the movement of food through the digestive tract, the digestive processes and the metabolic consequences of digestion. *See* text for details and references. Abbreviations: ca, caecum; cr, crop; DG, digestive gland; DT, digestive tract; st, stomach. Diagram of octopus and digestive tract modified from Gallardo et al., 2017.

It is important to evaluate the pH, temperature, substrate concentration and divalent ions that could specifically regulate digestive enzymes activity in each region of the tract (Martínez et al., 2011; Ibarra-García et al., 2018). Sampling requires the animal to be anaesthetized and killed (e.g., Roumbedakis et al., 2020). As soon as possible the digestive tract should be separated immediately into the crop, stomach, caecum and digestive gland using surgical clamps to avoid mixing the luminal contents. Once separated, the volume and weight of the chyme (the mixture of secretions [e.g., mucus and enzymes], partially digested food and ingested fluid) contained in each region should be measured, immediately frozen in liquid nitrogen, and stored until analysis (depending on the time until analysis, samples can be stored at −20, −40 or −80°C). A wealth of information on the digestive physiology of octopus can be obtained from this type of study particularly when combined with other techniques (e.g., *in vitro* studies of motility or preservation of tissue for IHC; *see* above); measurements are summarised in Figure 5.

In *O. maya, O. mimus* and *O. vulgaris* juveniles and adults, soluble nutrients (e.g., soluble protein, small peptides and amino acids) in the food move quickly to the DG and pass to the circulation becoming available for tissue metabolism, only 40–60 min after ingestion (Rosa et al., 2004; Linares et al., 2015).

The digestive physiology of octopus species and probably of other cephalopods can be divided into two phases: *1*)soluble nutrients pre-digested in the prey (or elaborated food) quickly pass-through the crop, stomach and caecum to the DG and then the haemolymph to be transported to tissues; *2*) more complex nutrients (e.g., myofibrillar proteins, complex lipids) are processed by the simultaneous action of the enzymes (from salivary glands and DG) when food is in the crop with mechanical degradation by concerted action of the crop and stomach, filtration in the caecum and finally entering the DG *via* the ducts as a suspension.

A recent histochemical study of the digestive tract of *O. vulgaris* detected no digestive enzyme presence in the digestive tract epithelium (Fernández-Gago et al., 2019) consistent with the view that chyme formation during the digestive process is predominantly by digestive enzymes secreted from DG with a minor contribution from salivary enzymes. This means that the changes occurring as food passes along the digestive tract are mainly because of the pulses of enzyme production in DG during the digestion phases previously described in cephalopods (Semmens, 2002; Martínez et al., 2011; Linares et al., 2015). The time to digest a meal depends on the species and ambient temperature. For example, in tropical and sub-tropical pre-adults of *O. maya* (26°C; 0.500 kg), *O. mimus* (14°C; 1.2 kg) (Linares et al., 2015) and *O. vulgaris* type II from Brazil (20.8°C) (Bastos et al., 2020) the complete digestive process takes 6–7 h, while in *Octopus cyanea* the entire digestive process was takes around 12 h at 30°C from the beginning of the meal (Boucher-Rodoni, 1973). Although digestion appears is a fast process in octopus species it is necessary to evaluate how temperature is modulating all the physiological, endocrinological and enzymatic mechanisms involved in the process to predict possible consequences in warming scenarios; a meta-analysis would be a useful first step.

### Measuring trans-epithelial absorption of material

Once the chyme is in the DG, biochemical process involving absorption and further digestion are activated (Boucaud-Camou and Boucher-Rodoni, 1983; Budelmann et al., 1997). In the DG of *O. maya* fasted for 24 h in laboratory conditions, “resting cells” characterised by few or no heterolysosomes or residual bodies are observed (Martínez et al., 2011). Additionally, an apocrine secretion and cell debris can be observed in the tubular lumen, probably resulting from catabolism in response to food deprivation for 24 h. During the first 1 and 2 h of the postprandial period (PP), digestive cells show nuclei forming a belt at the basal region of each columnar cell as the typical characteristic. Some heterolysosomes appeared inside the digestive cells. Acidophilic chyme in the tubule lumen indicates soluble nutrients in the DG. Once the digestive system in the DG is activated, heterophagosomes in the acinar cells transport amino acids to the haemolymph and then muscle and other tissues. In *O. maya*, *O. mimus* and *O. vulgaris* type II studies suggest that soluble protein is accumulated in muscle tissues initiating the glycosynthetic process (Gallardo et al., 2017; Bastos et al., 2020). After 4 h PP, the digestive cells store heterolysosomes (nutrients). Additionally, heterophagosomes were observed only near the apical zone of the cell, revealing the beginning of complex nutrient transport because of the mechanical digestion of more complex nutrients. Digestive cells showed a brush border (i.e., microvilli) on the apical membrane contacting the lumen. Acidophilic chyme was observed in the centre of the lumen. Peaks of glucose, cholesterol, acylglycerols and soluble protein can be observed with high digestive activity in DG cells. All these processes indicate that nutrients were transformed and transported to the tissues for energy and anabolic processes. At 6 h PP, the digestive cells have a conspicuous brush border and an empty lumen. Digestive cells appear filled with heterolysosomes, without heterophagosomes. At 8 h PP, a reduction in heterolysosomes was be observed, together with residual bodies, cell debris and apocrine secretion in the lumen. The proliferation of nuclei indicated the presence of replacement cells located in the basal lamina of the tubules signalling the end of the process (Boucaud-Camou and Boucher-Rodoni, 1983; Martínez et al., 2011; Gallardo et al., 2017; Fernández-Gago et al., 2019; Bastos et al., 2020).

### Measuring the energetic costs of food processing: specific dynamic action and apparent heat increment

Before describing the measurement of metabolic rate we first highlight one of the important parameters derived from its measurement. Many years ago it was recognized that the apparent heat increment (AHI) also termed specific dynamic action, (SDA) could be used as an indicator of the cost of mechanical and biochemical processes associated with the ingestion and assimilation of food (Nixon, 1969). Although muscular tissue is responsible for the mechanical activity, and the epithelium of the tract synthesises and secretes chitin and mucus requiring energy, the digestive gland is the main site of metabolic functions (Boucaud-Camou and Boucher-Rodoni, 1983). Hence, the AHI may result from addition of the energy used in the above processes; depending on the diet and temperature this constitutes a considerable percentage of the daily energy budget in aquatic organisms (Katsanevakis et al., 2005; Aguila et al., 2007; Noyola et al., 2013). In *O. vulgaris* values between 25 and 55% of food conversion efficiency were calculated. When different temperatures were compared it was observed that the peak value of AHI (SDA) of *O. vulgaris* was 64% at 20°C and 42% at 28°C (Katsanevakis et al., 2005) and in *O. cyanea* AHI was 60% of the ingested food (Nixon, 1969; Boucaud-Camou and Boucher-Rodoni, 1983). In *O. maya* it was also demonstrated that the AHI values are dependent on the type of diet with values that oscillate between 9 and 14% of the ingested energy when animals were fed fresh crabs or mixed diets made from fresh crab meat and other ingredients (Aguila et al., 2007; Rosas et al., 2007; Rosas et al., 2008). When temperature was investigated, the AHI values of *O. maya* were relatively higher in animals maintained in optimal temperature ranges (30–48% of routine metabolic rate; 22–26°C) than observed in animals maintained at 30°C (21%) suggesting that in higher temperatures the energy invested in mechanical and biochemical transformation of food is decreased due to additional costs associated with maintaining a higher metabolic rate at higher temperatures (Noyola et al., 2013). That means that depending on the species and its thermal tolerance temperatures beyond the optimal thermal range could affect the general physiological condition of the animals reducing the energy available to be invested in processing food. In larvae of coral reef, tropical fish it was observed that a temperature of 31.5°C does not affect the AHI (SDA) magnitude and duration suggesting that this species (*Amphiprion percula*) is well adapted to temperatures expected even in warming scenarios (McLeod and Clark, 2016).

### Measuring metabolic rate

There has been considerable debate about measurement of respiratory metabolism in aquatic animals in the light of new hypotheses related to global warming and physiological adaptation of ectotherms (Sokolova et al., 2012a; Sokolova et al., 2012b; Norin and Clark, 2016; Pörtner et al., 2017). Although the debate has focused on fish (Chabot et al., 2016a), there is a broad consensus on measurement of metabolism in aquatic organisms (Steffensen, 1989; Chabot et al., 2016b; Svendsen et al., 2016). Two types of respirometer have been used:

i) *Flow-through respirometry.* This measures oxygen consumption by quantifying the difference between inlet and outlet oxygen concentration and adjusting the flow of water through the respirometer to maintain a pre-set oxygen content difference. Oxygen consumption is then calculated as the product of water flow through the respirometer per unit time and the difference in oxygen concentration of the water entering and exiting the respirometer. In this respirometer the constant inflow of clean water reduces or eliminates the hypoxia, hypercapnia and nitrogenous waste issues associated with closed-system respirometry, but it introduces mixing and equilibration (washout) problems (Steffensen, 1989), although this can be corrected (Niimi, 1978).
ii) *Intermittent-flow respirometry.* This combines short measurement periods in a recirculating, but closed, respirometer. After some time (determined by the oxygen level in the respirometric chamber: not lower than dissolved oxygen saturation of 80%) the chamber is flushed with clean water to ensure that the water in the respirometer has been thoroughly exchanged to eliminate potential hypoxia, hypercapnia and nitrogenous waste build-up in the chamber (Steffensen, 1989). While this oxygen consumption method is the most popular amongst fish physiologists, it does require more equipment and a slightly more complex experimental setup than the flow-through respirometer (Svendsen et al., 2016). Although both methods have pros and cons, the most important aspect is the accuracy and validity of the measurements.

The development of oxygen sensors to measure dissolved oxygen facilitated experimental work in this area. Although polarographic oxygen probes are still used, optical sensors are considered the best to use in physiological evaluations of metabolic rate of aquatic animals including cephalopods. Much of the data obtained in the last 50 years on octopus species used the home tank as a respirometric chamber and in the many cases as closed respirometers (Wells et al., 1983; O'Dor and Wells, 1987; Wells et al., 1996; Cerezo Valverde and Garcia Garcia, 2005; Katsanevakis et al., 2005). Although those data give useful information about the respiratory physiology of several octopus species, using optical sensors and improving the systems (i.e., Intermittent-flow or flow-through respirometry) will give more precise data as shown in *O. vulgaris* and *O. maya* (Cerezo Valverde and Garcia Garcia, 2004; Aguila et al., 2007; Noyola et al., 2013; Martínez et al., 2014; Meza-Buendía et al., 2021).

Data on oxygen consumption can be used to evaluate the energetic costs of different types of food (natural and elaborated) if the AHI is measured but also, if ingested food (I), faeces production (F), nitrogen excretion (N) and growth (P) are measured (all expressed in energy units; J day^−1^ g^−1^ or kg^−1^), these values can be integrated into an energetic flow equation (Wells et al., 1996):

I = F + N + P + R

where assimilated energy (As) can be calculated as:

As = R + P

In this equation R = R_AHI_ + R_rut_, with R_rut_ the metabolic rate of animals before feeding in respirometric chamber. Studies made in *O. maya* show stable R_rut_ values can be obtained in animals conditioned to respirometric chambers for 12–18 h, depending on temperature and the animal size (Roumbedakis et al., 2020; Meza-Buendía et al., 2021). When this type of data is obtained in animals fed different types of food it is possible to know how the nutritional characteristics of the food modulate the quantity of energy that is channelled to growth in comparison to mechanical and biochemical transformation of the ingested food. When pre-adult *O. maya* were fed *Callinectes sapidus* crab, R was 30% of As, but animals fed with elaborated food made from fish meal the R/As, % was between 80 and 90% indicating that a significantly proportion of the ingested energy was used to transform that diet in physiologically useful energy reducing the energy available for growth (Aguila et al., 2007). When an elaborated paste made from crab and squid was used to feed early juveniles of this species, a R/As value of 27%, was obtained. Analysis of R/A% and growth shows that lower values (∼30%) are associated with higher growth rates but diets with high R/A% (80–95%) are associated with lower growth as almost all the ingested energy is channelled to maintenance but not growth (Van Heukelem, 1976; Martínez et al., 2014).

### Omics and molecular biology

The utility of molecular studies is well illustrated by studies investigating effects of the gastrointestinal parasite *Aggregata octopiana*. Changes in gene expression in tissue from discrete regions of the DS provides insights into physiology. For example, in the octopus gastric ganglion expression of selected genes correlated with increased relative expression of six genes in conditions of high infection (i.e., Sn, Nfkb2, Cckar, SCPRP, Serpinb10, Tlr3) while reduced expression was reported for others (e.g., Litaf, Mirp, Sod1, Prdx6, Gpx1, Rph3al) (Baldascino et al., 2017). The study demonstrated effects of the parasite on a neural structure at a site distant from the locus of infection in the intestinal wall. Transcriptomic analysis of haemocytes identified >500 differentially expressed transcripts in *Aggregata* infected octopus infected with *Aggregata* including genes involved in pathways such as Nuclear Factor-kB, Toll-like receptor, and Complement (e.g., Castellanos-Martínez et al., 2014) or transcriptional variations of diet, temperature, and growth-related genes in paralarvae (e.g., García-Fernández et al., 2019; Garcia De La Serrana et al., 2020).

Proteomics further characterised salivary glands and their secretions. For example, the Southern blue-ringed octopus (*Hapalochlaena maculosa*) is a remarkable exception with loss of proteinaceous toxins due to the presence of tetrodotoxin (Whitelaw et al., 2020). The gland has >600 genes exclusive to *H. maculosa* when compared to *O. bimaculoides* and *Callistoctopus minor*) providing an overall scenario of fewer and more specialized genes expressed by other octopod species, when compared to *Hapalochlaena.*

Although omics and molecular studies provide novel insights it is important that findings are translated where possible into understanding the physiology of the digestive system.

### Investigating neural and endocrine control mechanisms

Relatively little is known about how the individual functions described above are controlled, coordinated with each other (e.g., the relationship between the crop, stomach and caecum and the secretory and absorptive phases of DG activity) and with the regulation of food intake. Here we briefly review selected studies to illustrate some of the applicable techniques.

#### Neural control

a) *The enteric nervous system (ENS).* The ENS comprises the neurones within the wall of the digestive tract which may act as relays between the gastric ganglion and the muscle/epithelium to modulate secretion and motility but which also form independent reflex circuits comprising intrinsic afferent (the subepithelial plexus is reported to consist mainly of sensory cells in cuttlefish, Alexandrowicz, 1928) and efferent neurones (Furness and Stebbing, 2018). Although there is good histological evidence for the presence of intramural neurones throughout the digestive tract in cephalopods (e.g., Alexandrowicz, 1928; Young, 1967) only a limited number of species have been studied, with limited techniques (e.g., no tract tracing studies to define the exact relationship to the GG or immunohistochemistry) and there are no specific functional studies. Involvement of ENS in coordinated peristalsis and mucus secretion is highly probable by analogy with other species (Costa and Furness, 1976; Furness, 2006) as is modulation by extrinsic nerves (e.g., from the gastric ganglion) but these speculations await experimental confirmation using for example *in vitro* pharmacological studies (*see* above), and neurophysiological recordings from enteric neurones as have been performed extensively in mammals (Furness, 2006).
b) *The gastric ganglion (GG).* The structure and neurochemical complexity of the GG is consistent with its proposed role as a peripheral integrative centre but limited studies have only been undertaken in *O. vulgaris.* Electrical stimulation of the GG in *O. vulgaris* increased contractile activity in the crop, stomach, caecum (spiral tightening) and proximal intestine (Andrews and Tansey, 1983a) and there is a single report (Falloise, 1906) of accelerated flow from the digestive gland which could be due to capsule contraction, expulsion of stored secretion or *de novo* synthesised secretions. However, there have been no neurophysiological studies demonstrating the integrative abilities of the GG, the properties of its neurones or how it interfaces with the ENS, although its compact nature and structure (cortical layer of cells) make it ideally suited for these types of study. Neurophysiological studies of the stomatogastric ganglion in other molluscs (e.g., Jing et al., 2007; Daur et al., 2016) provide examples of the techniques and types of study which need to be undertaken.

Although surgical removal of the GG would be a technically simple procedure, it is unlikely to yield results which would be readily interpretable because of its extensive influence. *In vitro* studies of the entire digestive tract in *O. vulgaris* showed that GG removal/local anaesthetic application disrupted coordination of contractions between the crop and stomach and also contracted the crop suggesting removal of a tonic inhibitory effect of the GG on the crop (Andrews and Tansey, 1983a).

Molecular and immunohistochemical studies of the *O. vulgaris* GG (Baldascino et al., 2017) have identified a number of putative peptide neurotransmitters (e.g., cephalotocin, corticotrophin releasing factor, FMRF-amide, octopressin, small cardioactive peptide-related peptide, tachykinin related peptide) and receptors (e.g., cholecystokinin _A,B_, octopressin, orexin receptor_2_). However, functional studies are limited to showing contraction of the radula muscle (small cardioactive peptide-related peptide tachykinin related peptide, Kanda and Minakata, 2006) and contraction of the crop and stomach (tachykinin related peptide, Kanda et al., 2007) or rectum (octopressin, Takuwa-Kuroda et al., 2003). Detailed studies of the effect of putative peptide neurotransmitters on digestive tract motility and secretions are needed both by investigating their direct effects (*in vitro studies*) and also indirect effects by acting on the GG where they could induce motor program switching (e.g., Kirby and Nusbaum, 2007).

The role of the GG in regulating individual regions of the digestive tract could also be investigated by selective surgical transection (under general anaesthesia, with recovery and including a sham lesion group) of nerves radiating from the ganglion to adjacent structures. Following recovery, urge to eat, food intake, growth, transit time, faecal composition, haemolymph composition and post-prandial metabolism (O_2_ consumption) could be measured to identify the impact of the lesion. Such a study would need careful justification, with clearly defined outcomes. Interpretation of the results may be complicated as the sectioned nerves may contain both afferent and efferent fibres and digestion of food is a sequential process with interdependent components.

c) *The central nervous system (CNS), interchange of information with the digestive system, and control of food intake*.

Communication (neural and/or endocrine) between the digestive system and the CNS is essential for the regulation of food intake which requires behaviour change to locate, capture and ingest food at a time when digestion of the previous meal permits. Limited evidence from changes in attack behaviour in *O. vulgaris* at different times after feeding has been used to conclude that the crop sends information on its degree of distension to the CNS (directly or *via* the GG; *see*: Young, 1960; Nixon, 1965). Additionally, as not all nerve fibres degenerate when the sympathetic nerves are cut between the inferior buccal ganglion and oesophagus it is concluded that the nerve contains some afferent fibres (Young, 1965). Neurophysiological studies are required to record from the sympathetic nerves to investigate whether they convey information about crop distension to the CNS in octopus or in the case of cuttlefish and squid from the stomach. If mechanosensitive afferents are present, we hypothesise by analogy with mechanoreceptors in hollow visceral organs of vertebrates, that they will be in-series with the muscle and hence capable of signalling both overall level of distension and contractions (*see* for example Andrews et al., 1980).

A detailed neurophysiological survey of the information carried in the nerves connecting the GG to the DT, the visceral nerves, and the sympathetic nerves connecting with the inferior buccal ganglion and superior buccal lobe is required to understand the relative role(s) played by the CNS, GG and ENS.

The effects of surgical transection of the nerves connecting the CNS and digestive system was investigated by Best and Wells (1983) in *O. vulgaris* but the observations have not been pursued. In food deprived animals the sight of a crab in a sealed glass jar stimulated secretion in the DG, an effect prevented by section of the sympathetic nerves either above or below the crop implying a response driven by the CNS. This is the equivalent of the “cephalic phase” of digestive tract secretions first reported by Pavlov in dogs (Pavlov, 1897). However, the majority of animals (16/18) with sympathetic nerves cut were unable to clean crabs after successfully attacking complicating interpretation. Section of the abdominal and atrio-rectal branches of the visceral nerve was without apparent effect on the ability to clean a crab, the DG response, post-prandial rise in O_2_ consumption or growth. Although representing unique observations the results should be treated with caution until replicated using more refined surgical techniques.

#### Endocrine control

The control of the DS by substances acting *via* the haemolymph has not been investigated in any detail especially in comparison to the reproductive system in cephalopods (e.g., Zatylny-Gaudin et al., 2016). Hormones synthesised outside the digestive system could regulate any of the DT functions directly or *via* the GG but also the potential for hormones released from the digestive tract to act on the GG or on the CNS for example to regulate food intake should not be overlooked. With the possible exception of the posterior salivary glands (Ponte and Modica, 2017) histological studies have not identified cells with characteristics typical of endocrine or paracrine cells in the digestive system but there have not been any systematic studies. Measurements of haemolymph during food deprivation and post-prandially would be the most obvious approach to identifying hormones linked to digestion with candidates identified from analysis of the genome. Based particularly on molecular studies, the following families are of particular interest: *1*) *secretin* (Cardoso et al., 2010; Mirabeau and Joly, 2013; Tam et al., 2014). A secretin-like substance was isolated from the caecum in *O. vulgaris* and shown to stimulate secretion in the digestive gland (Ledrut and Ungar, 1937). This study should be followed up as a major function of secretin is stimulation of fluid secretion in the vertebrate exocrine pancreas (Bayliss and Starling, 1902); *2*) *vasopressin/oxytocin* (Odekunle et al., 2019). Evidence for the octopressin receptor in the GG has been obtained (Baldascino et al., 2017) and Odekunle et al. (2019) noted evidence implicating the vasopressin/oxytocin -type signalling from a number of studies of protostomes; *3*) gastrin/cholecystokinin (Zatylny-Gaudin and Favrel, 2014) as there is evidence for cholecystokinin _A,B_ receptors in the GG (Baldascino et al., 2017); *5*) *orexin* (Ritschard et al., 2019). There is limited evidence for the orexin receptor_2_ in the GG but the ligand in cephalopods is not known although members of the orexin family have been implicated in regulation of food intake in vertebrates (Wong et al., 2011).

Molecular and haemolymph profile studies need to be complemented by studies showing a functional effect of any hormones on the digestive tract. In addition, the effect of reproductive hormones on the digestive tract needs to be investigated as they may be involved in the suppression of food intake which accompanies egg laying and care in several octopus species (e.g., *O. maya*).

## Concluding comments

**FIGURE 6:**
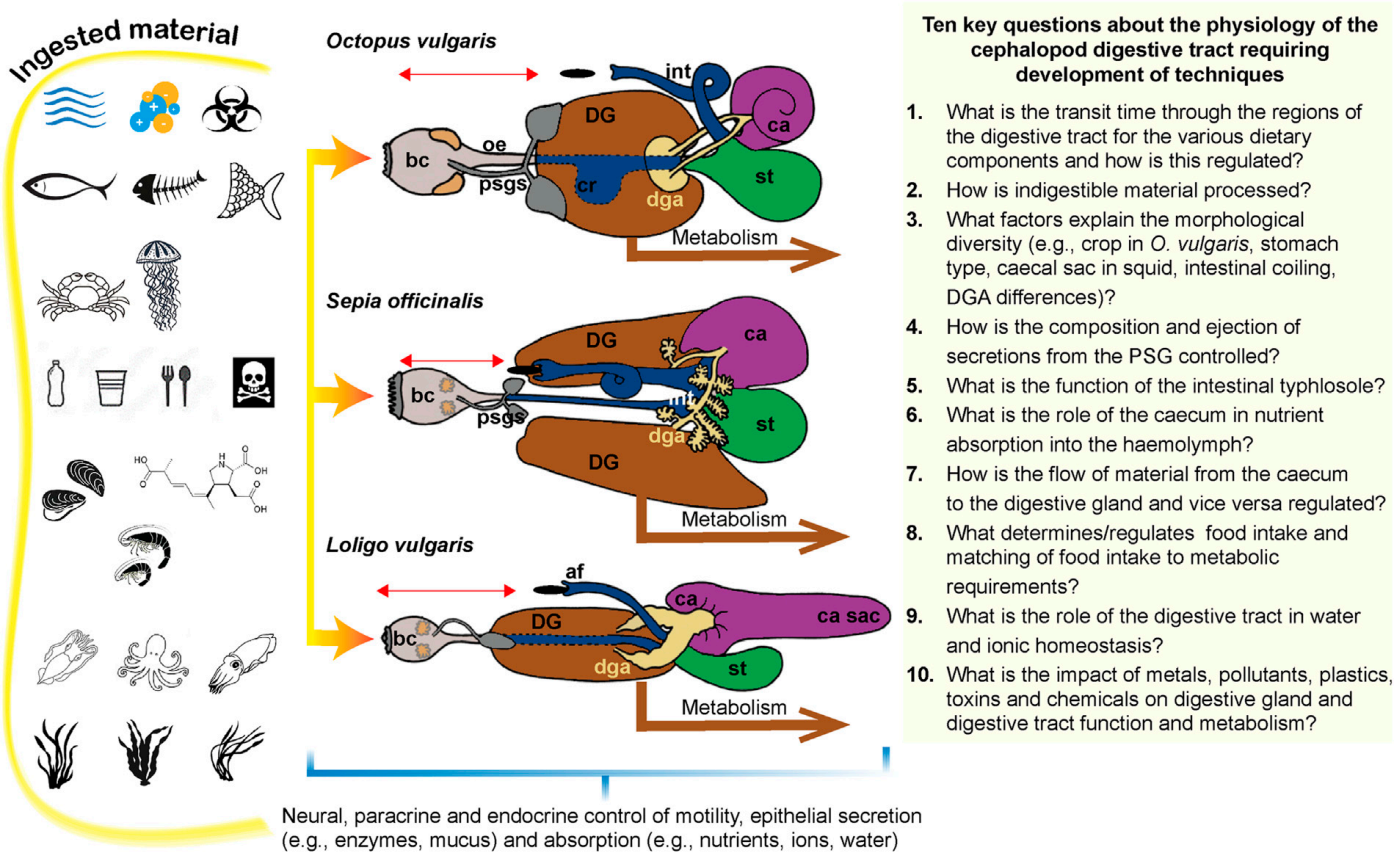
Diagram summarising a number of the key issues discussed in the review. The left -hand panel shows some of the diverse range of materials (food and non-food items) which can enter the digestive tract of cephalopods and which it will need to digest/detoxify/metabolise or eject (vomiting or defaecation). From left to right in each row the symbols indicate: water (may also contain dispersed oils), ions, biohazardous material, fish (or pieces), fish bones, fish scales, crustacea, jellyfish, plastic (entire items or fragments depending on animal size), toxins (may be adsorbed to plastics), mussels and other shellfish, domoic acid (may be present in mussels), shrimps, cephalopods (including conspecifics), seaweed and seagrasses (probably incidentally ingested with other food). The middle panel shows gross anatomical features of the digestive tract of an exemplar octopus, cuttlefish and squid to highlight the major gross anatomical differences. The horizontal red line represents the transit time-the time taken from when food enters the digestive tract *via* the beak to when the remains of that meal leave in the faeces (black oval). Abbreviations: af, anal flaps; bc, buccal complex; ca, caecum; cr, crop; DG, digestive gland; dga, digestive gland appendage; int, intestine; oe, oesophagus; psgs, posterior salivary glands; st, stomach. The right -hand panel list 10 key questions which need to be answered about the physiology of the cephalopod digestive tract and which will require the application of some new techniques and development of other to investigate in cephalopods. *See* text for detailed discussion references. The diagram in the middle panel is modified from Lobo-da-Cunha, 2019.

We have reviewed diverse techniques applied to understanding the physiology of the digestive tract which enable it to perform its primary function of processing food into a form which can be utilized. Three themes emerge: *1*) Many of the pivotal studies were published more than 50 years ago and should be replicated using more modern techniques to both confirm and extend the initial findings; *2*) There are a number of major knowledge gaps (Figure 6) (e.g., transepithelial transport in the caecum/intestine) which can be resolved using established physiological techniques (e.g., Ussing chamber, molecular identification of transporters) and assumptions about function are made based primarily upon anatomy (e.g., typhlosole function); *3*) Our understanding of the physiology of the digestive tract in cephalopods is a “mosaic of knowledge” with data on individual DT functions coming from a range of species in different Orders rather than a detailed understanding of the entire digestive tract in representative species (e.g*.*, *O. vulgaris* and *S. officinalis*). As Bidder (1950, p. 41) noted 70 years ago “Until more information is available it is clearly dangerous to combine, as has too often been done, one observation from *Octopus* with another from *Sepia* under the generalisation “In the Cephalopods.””

It is hoped that this review by combining discussion of the physiology of the cephalopod DS with an overview of techniques and identification of key knowledge gaps will stimulate a more systematic approach to research in this area.
